# Colony Collapse Disorder (CCD) and bee age impact honey bee pathophysiology

**DOI:** 10.1371/journal.pone.0179535

**Published:** 2017-07-17

**Authors:** Dennis vanEngelsdorp, Kirsten S. Traynor, Michael Andree, Elinor M. Lichtenberg, Yanping Chen, Claude Saegerman, Diana L. Cox-Foster

**Affiliations:** 1 Department of Entomology, Plant Science Building University of Maryland, College Park, Maryland, United States of America; 2 Cooperative Extension Butte County, University of California Cooperative Extension, Oroville, California, United States of America; 3 Department of Ecology and Evolutionary Biology, The University of Arizona, Tucson, Arizona, United States of America; 4 USDA-ARS, Bee Research Laboratory, Beltsville, Maryland, United States of America; 5 Research Unit in Epidemiology and Risk Analysis applied to Veterinary Sciences (UREAR-ULg), Fundamental and Applied Research for Animal and Health (FARAH) Center, Faculty of Veterinary Medicine, University of Liège, Liège, Sart-Tilman, Belgium; 6 USDA-ARS-PWA, Pollinating Insect-Biol., Mgmt. Syst.- Research Unit, Logan, Utah, United States of America and Department of Entomology, The Pennsylvania State University, University Park, Pennsylvania, United States of America; Ghent University, BELGIUM

## Abstract

Honey bee (*Apis mellifera*) colonies continue to experience high annual losses that remain poorly explained. Numerous interacting factors have been linked to colony declines. Understanding the pathways linking pathophysiology with symptoms is an important step in understanding the mechanisms of disease. In this study we examined the specific pathologies associated with honey bees collected from colonies suffering from Colony Collapse Disorder (CCD) and compared these with bees collected from apparently healthy colonies. We identified a set of pathological physical characteristics that occurred at different rates in CCD diagnosed colonies prior to their collapse: rectum distension, Malpighian tubule iridescence, fecal matter consistency, rectal enteroliths (hard concretions), and venom sac color. The multiple differences in rectum symptomology in bees from CCD apiaries and colonies suggest effected bees had trouble regulating water. To ensure that pathologies we found associated with CCD were indeed pathologies and not due to normal changes in physical appearances that occur as an adult bee ages (CCD colonies are assumed to be composed mostly of young bees), we documented the changes in bees of different ages taken from healthy colonies. We found that young bees had much greater incidences of white nodules than older cohorts. Prevalent in newly-emerged bees, these white nodules or cellular encapsulations indicate an active immune response. Comparing the two sets of characteristics, we determined a subset of pathologies that reliably predict CCD status rather than bee age (fecal matter consistency, rectal distension size, rectal enteroliths and Malpighian tubule iridescence) and that may serve as biomarkers for colony health. In addition, these pathologies suggest that CCD bees are experiencing disrupted excretory physiology. Our identification of these symptoms is an important first step in understanding the physiological pathways that underlie CCD and factors impacting bee health.

## Introduction

Honey bee (*Apis mellifera*) health has been in decline, with winter losses in the United States averaging 30% since Colony Collapse Disorder (CCD) was first identified in 2006 and annual losses approaching 50% [1–8]. Numerous interacting factors have been implicated in colony declines including poor nutrition, increased pressure from ecto- and endoparasites, increased bacterial and/or viral loads, and synergistic pesticide interactions [9–13]. Failure to recognize the early symptoms of disease in honey bee colonies could hamper the treatment and containment. The first stages of honey bee colony decline often go unrecognized, as healthy colonies have excess populations, which act as buffers against sudden losses in the work force [14,15]. When large numbers of the older cohort of forager bees die, younger in-hive bees accelerate maturation and become precocious foragers to replace foraging work force losses[16]. The subtle changes in a colony’s population, as a result, are hard to detect, meaning that beekeeper intervention may not occur until the colony is already weak or has died. An ability to look at adult honey bee anatomical and physiological characteristics to predict poor honey bee health could allow for early disease detection, enabling timely intervention.

Pathophysiology, an approach that studies the structural and functional changes in tissues that accompany disease, is a valuable tool for early detection of disease in human subjects [17,18]and may also have potential application when monitoring honey bee health. Linking physiological symptoms with a disease can elucidate the pathways in dysfunction that culminate in disease outbreak and/or symptomology[19]. Because managed pollinators frequently don’t show external indications of ill health until they are near death, the use of pathophysiology has particular promise towards predicting and understanding bee disease in general and CCD specifically. The U.S. experienced elevated colony losses in 2006–2008, many of which were characterized by a common set of specific symptoms: (1) the rapid loss of adult worker bees from affected beehives, resulting in weak or dead colonies with excess brood populations relative to adult bee populations; (2) a noticeable lack of dead worker bees both within and surrounding the affected hives; and (3) the delayed invasion of hive pests (e.g., small hive beetles and wax moths) and cleptoparasitism from neighboring honey bee colonies. Subsequently, this syndrome was termed CCD, and its case definition was revised to include (4) the absence of *Varroa destructor* and *Nosema* spp. loads at levels that cause economic damage [20]. Though affected colonies shared this specific set of symptoms, no single variable has emerged as the most likely cause of CCD. The bees do often have relatively high pathogen loads, suggesting an increased exposure or a weakened immune response [8,21–24].

For insects, the diagnosis of pathogens and a disease state begins with dissection to examine the internal tissues and structures for abnormal pathologies [25]. For all insects, preserving as much of the tissue without degradation is important, thus freezing and transferring the cadavers to 70% ethanol permits short term storage and dissection at a later date. For honey bees, many references and manuals have been created on how to diagnose pathogens and pests that might be found in the colony [26–30]. Most of these manuals discuss commonly found pests and pathogens that are diagnosed easily by external examination of the brood and adult bees. For tracheal mites and *Nosema*, methods for diagnosis require the dissection of adult bees [31–33]\. To perform these dissections, a good understanding of bee internal anatomy is required; for *Apis mellifera*, the anatomy is well described [34,35]and methods on how to perform dissections outlined [36]. To date, these methods have not allowed for the diagnosis of complex pathologies or disease states such as CCD. This manuscript puts forward methods using dissections with dissecting microscopes to find pathologies and their descriptions. These pathologies were described both for adult bees of different ages in healthy colonies and also for adult bees from collapsing colonies and bees in apiaries with CCD. Previous research has found that colonies located near collapsing colonies are at high risk of dying [20].

Here we apply pathophysiology to honey bees to identify gross abnormalities that may be useful in diagnosing colonies before they develop the late-stage disease symptoms typical of CCD. We first developed a standard ranking system to score the gross lesions and other symptoms seen in individual honey bees. We then statistically analyzed these symptoms and their distributions using a CART analysis and found differences in symptom frequencies between CCD and non-CCD bees. We remained concerned that these differences were due to bee age, as typically only young bees remain behind in colonies collapsing from CCD [20,37]. To determine which differences could be explained solely by age, we compared gross symptoms in bees performing different age-related tasks. In the end, we developed a list of physio-pathological traits that serve as biomarkers to predict colony health.

## Results

### Development of standard scoring system for lesions

**Fig 1:**
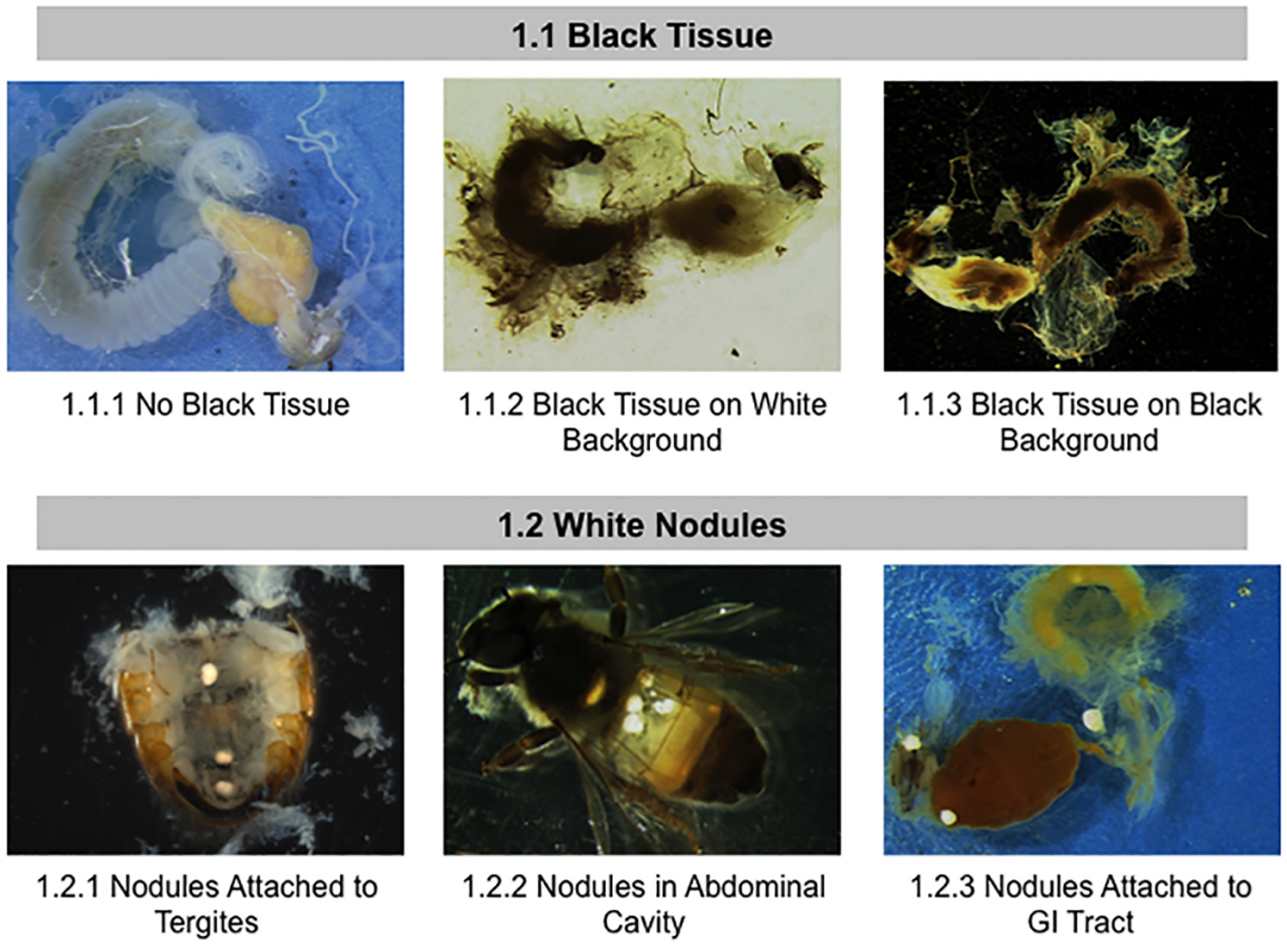
Normal and abnormal observed tissues in whole body of healthy bees and bees taken from CCD colonies. Location of the tissues and symptoms are described in the methods. These symptoms were used to the standardized scoring listed in Table 1. 1.1) Black Tissue in body; 1.2 White Nodules.

**Fig 2:**
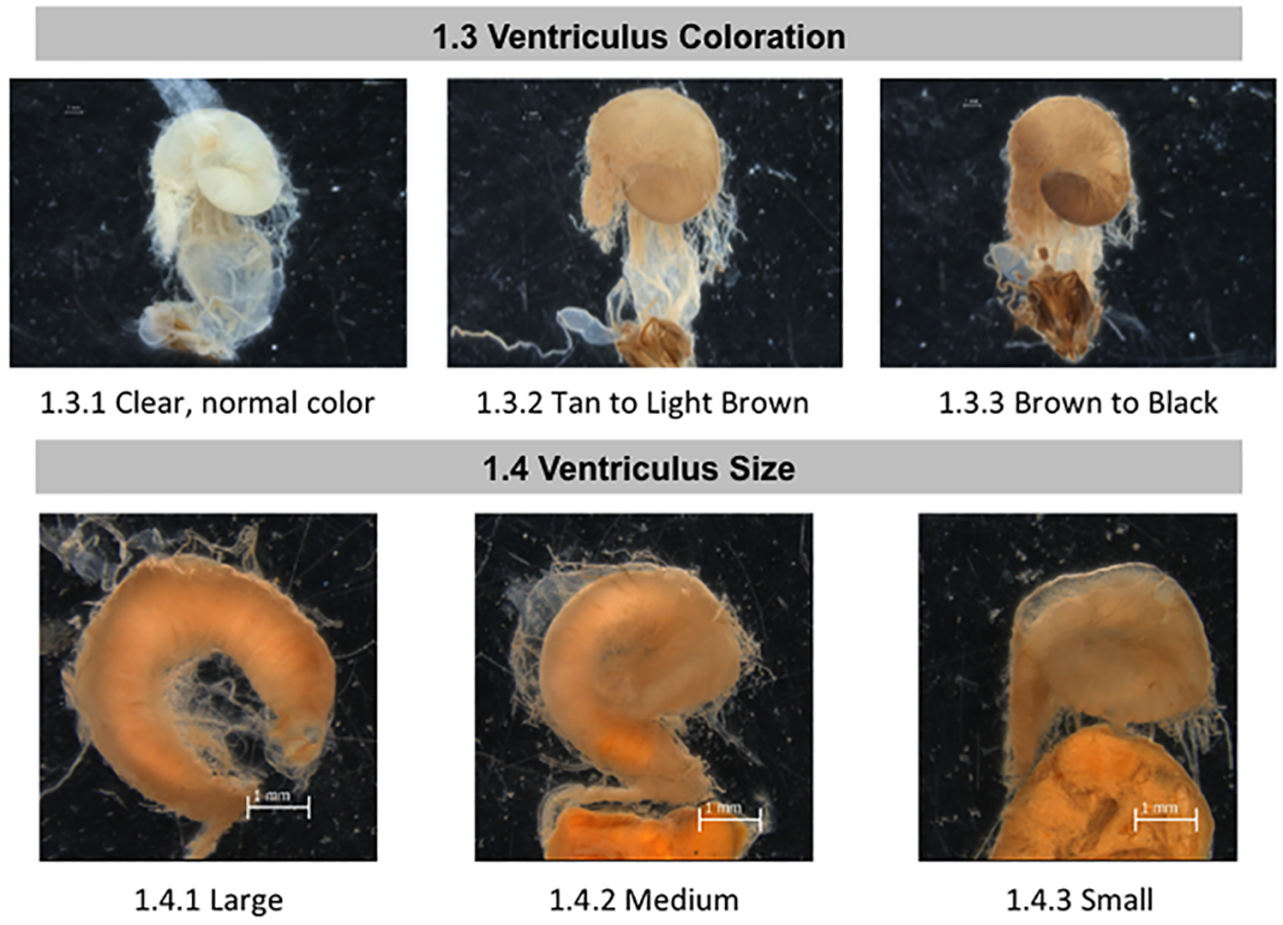
Normal and abnormal observed tissues in midguts of healthy bees and bees taken from CCD colonies. Location of the tissues and symptoms are described in the methods. These symptoms were used to the standardized scoring listed in Table 1. 1.3 Ventriculus Coloration; 1.4 Ventriculus Size.

**Fig 3:**
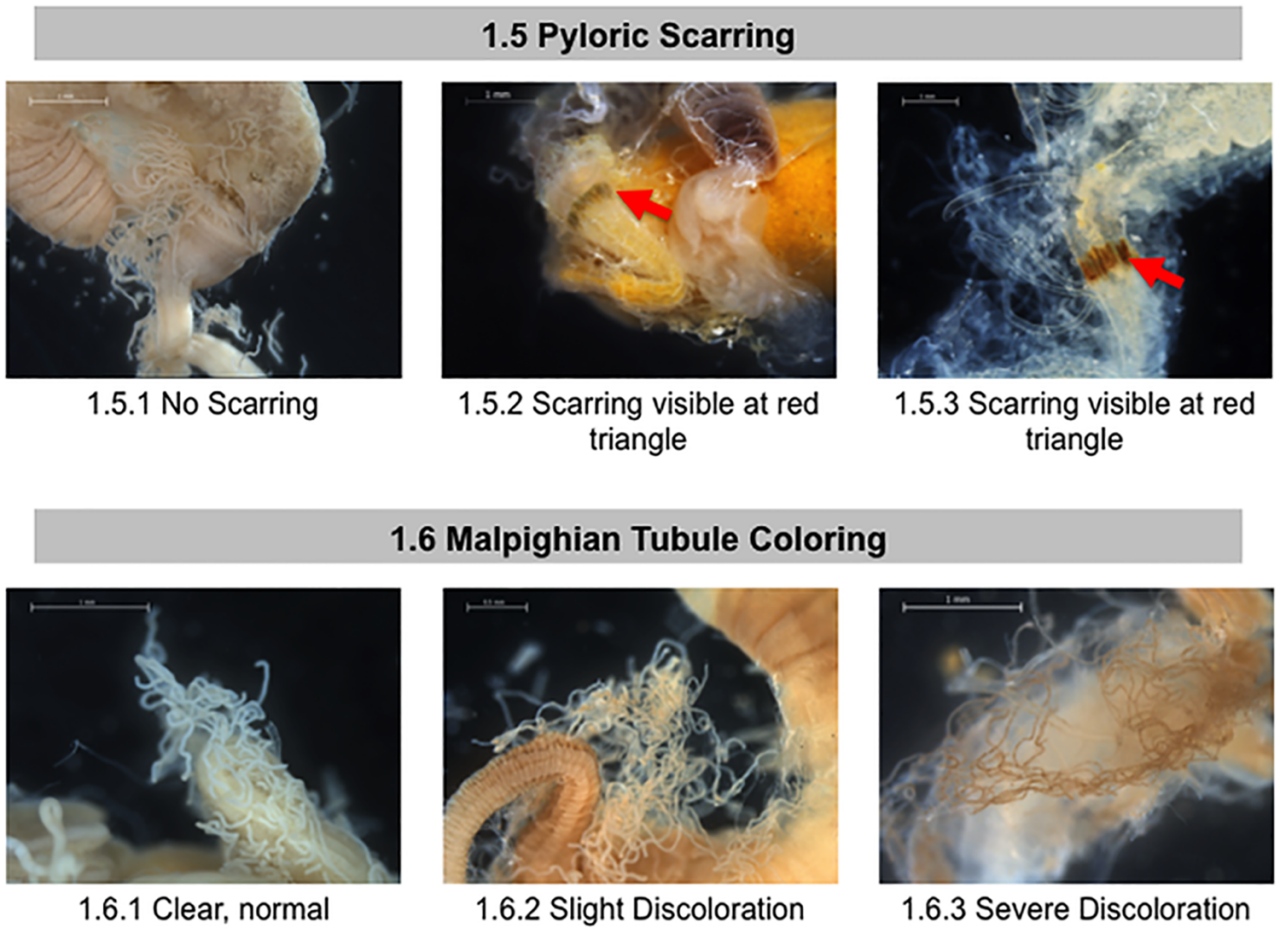
Normal and abnormal observed tissues in the pylorus and Malpighian tubules of healthy bees and bees taken from CCD colonies. Location of the tissues and symptoms are described in the methods. These symptoms were used to the standardized scoring listed in Table 1. 1.5 Pyloric Scarring; 1.6 Malpighian Tubule Coloring. Red arrows = phyloric scarring.

**Fig 4:**
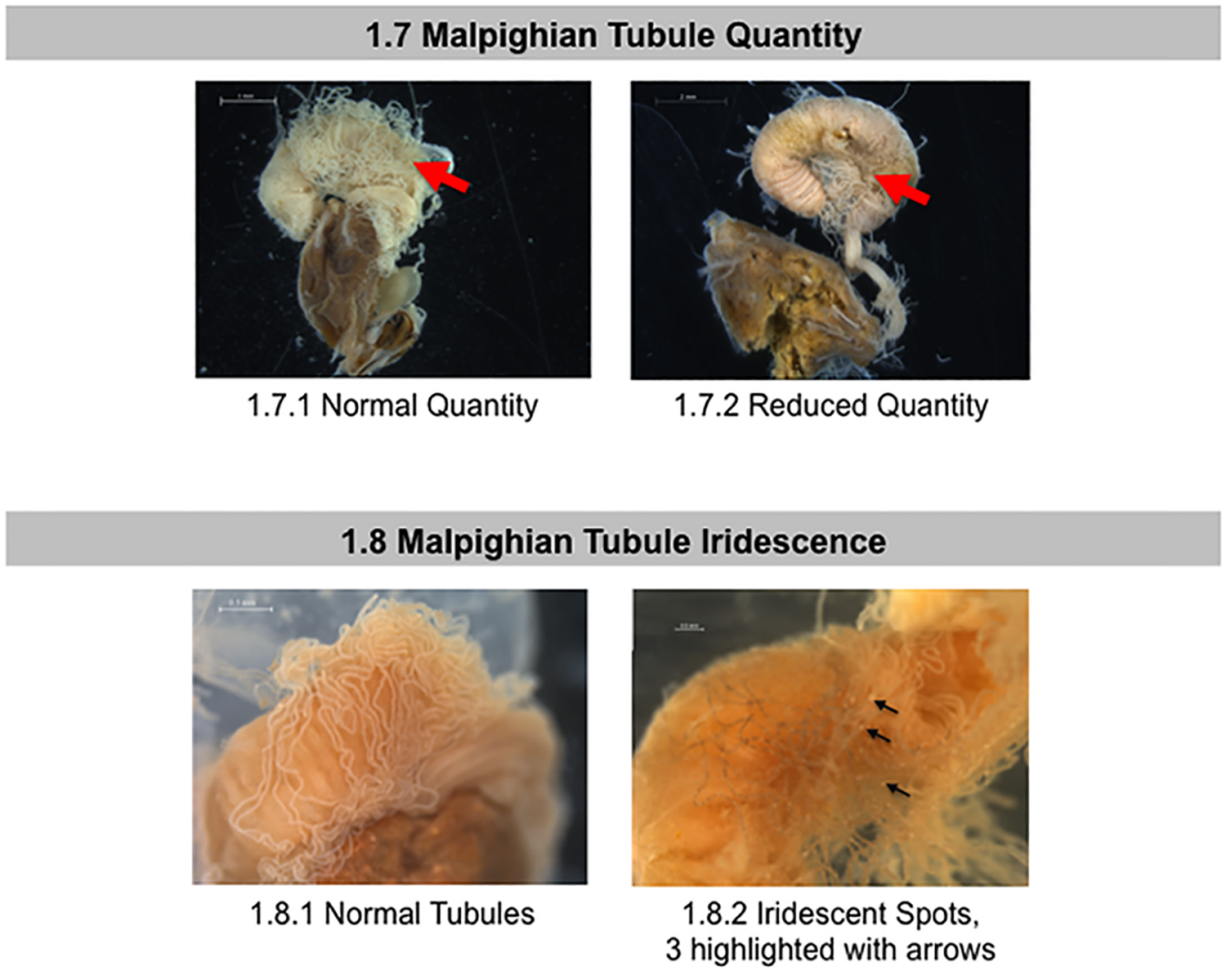
Normal and abnormal observed Malpighian tubules of healthy bees and bees taken from CCD colonies. Location of the tissues and symptoms are described in the methods. These symptoms were used to the standardized scoring listed in Table 1. 1.7 Malpighian Tubule Quantity; 1.8 Malpighian Tubule Iridescence. Red arrow = Malpighian tubules. Black arrows = concretions and iridescent spots on Malpighian tubules.

**Fig 5:**
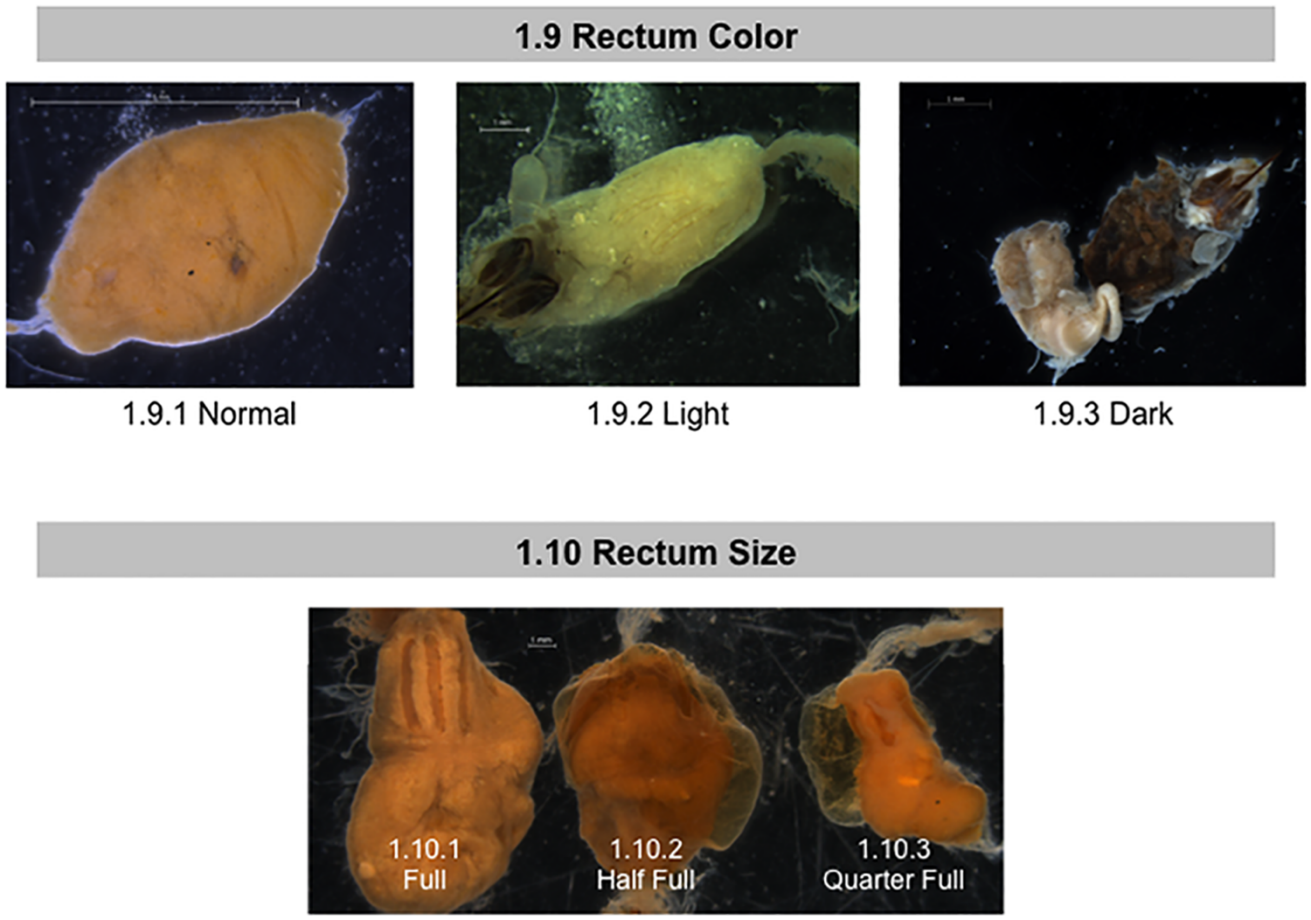
Normal and abnormal observed rectums of healthy bees and bees taken from CCD colonies. Location of the tissues and symptoms are described in the methods. These symptoms were used to the standardized scoring listed in Table 1. 1.9 Rectum Color; 1.10 Rectum Size.

**Fig 6:**
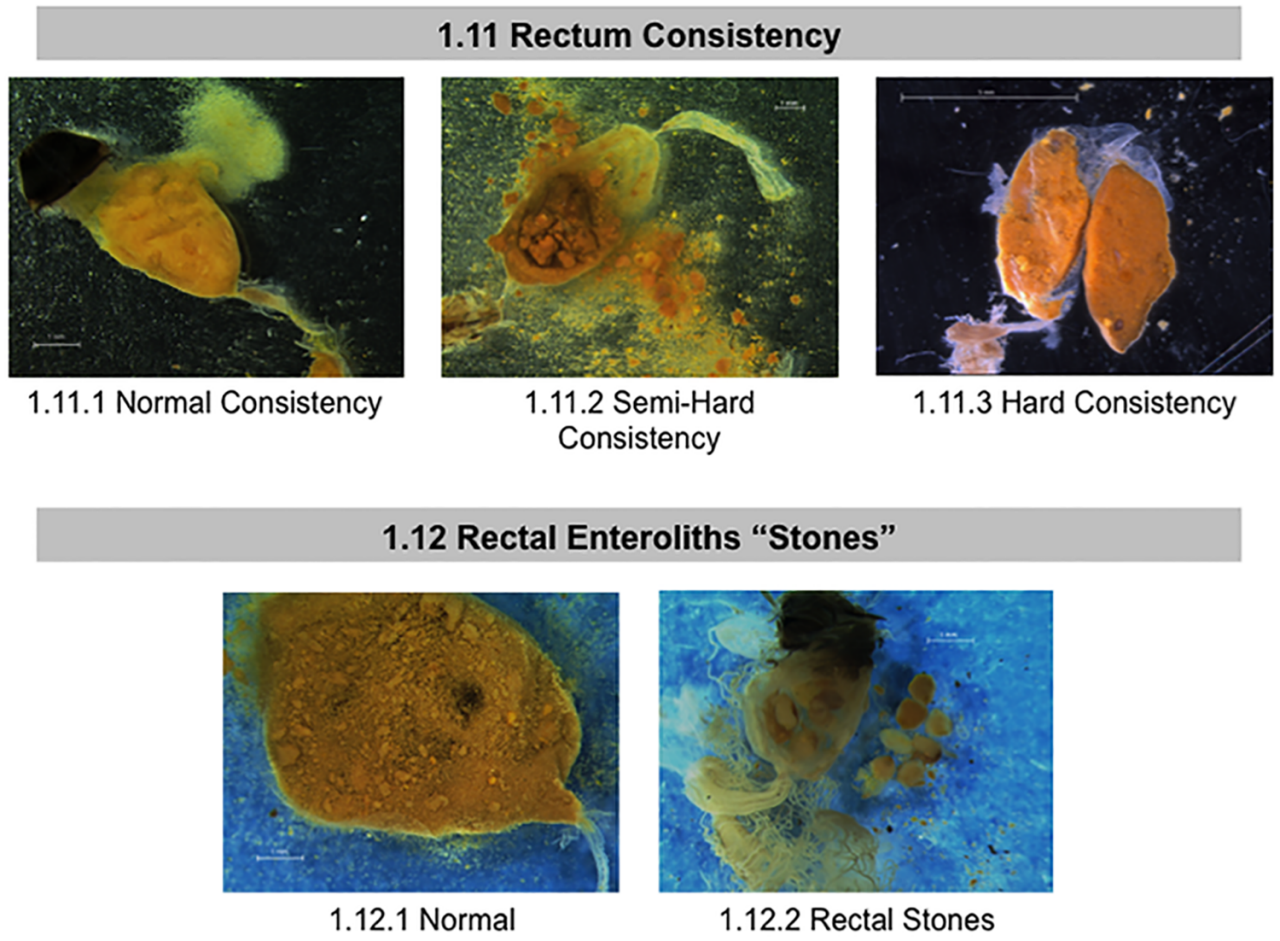
Normal and abnormal observed fecal contents of healthy bees and bees taken from CCD colonies. Location of the tissues and symptoms are described in the methods. These symptoms were used to the standardized scoring listed in Table 1. 1.11 Rectum Consistency; 1.12 Rectal Enteroliths “Stones”.

**Fig 7:**
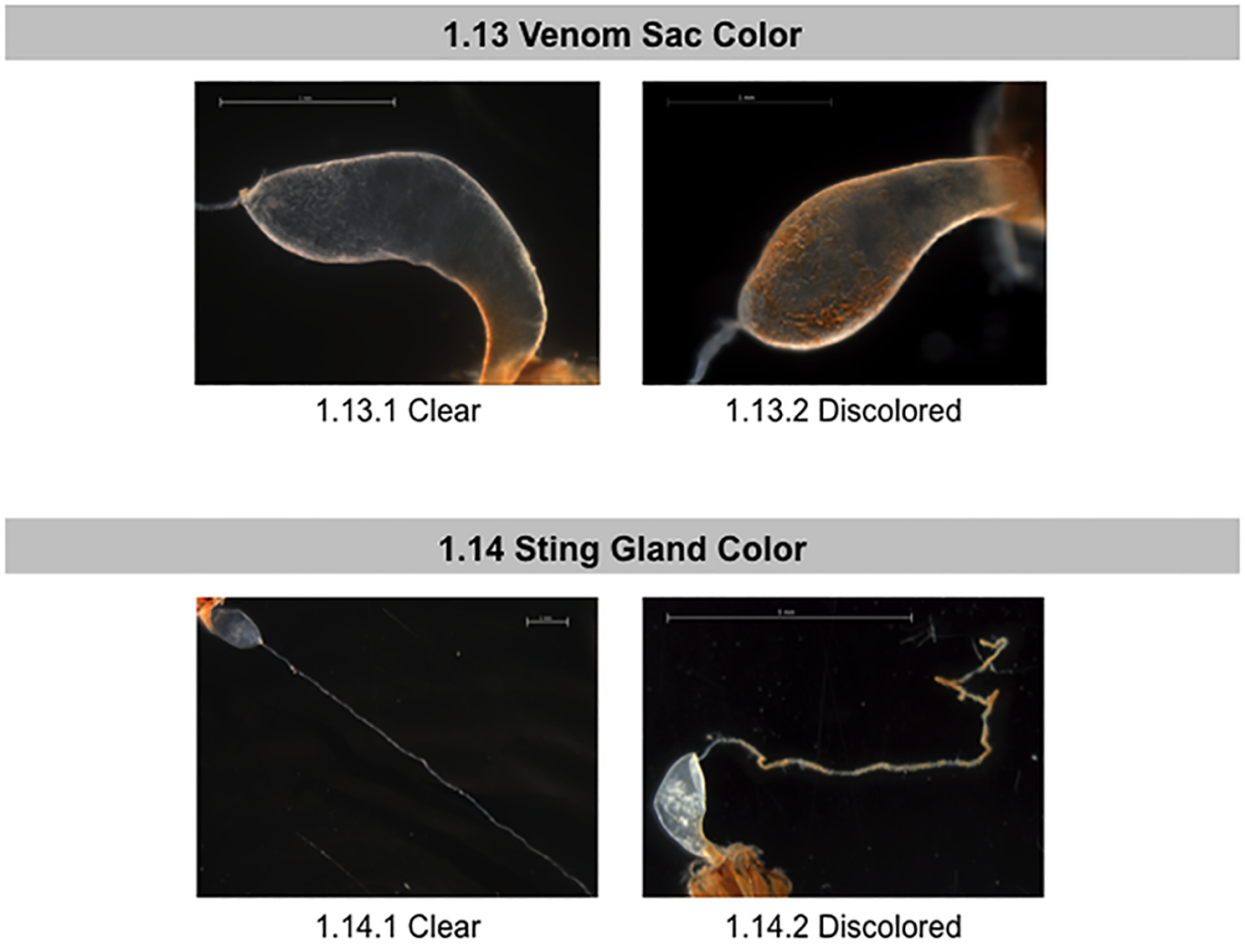
Normal and abnormal observed tissues in the venom sac and sting glands of healthy bees and bees taken from CCD colonies. Location of the tissues and symptoms are described in the methods. These symptoms were used to the standardized scoring listed in Table 1. 1.13 Venom Sac Color; 1.14 Sting Gland Color.

**Fig 8:**
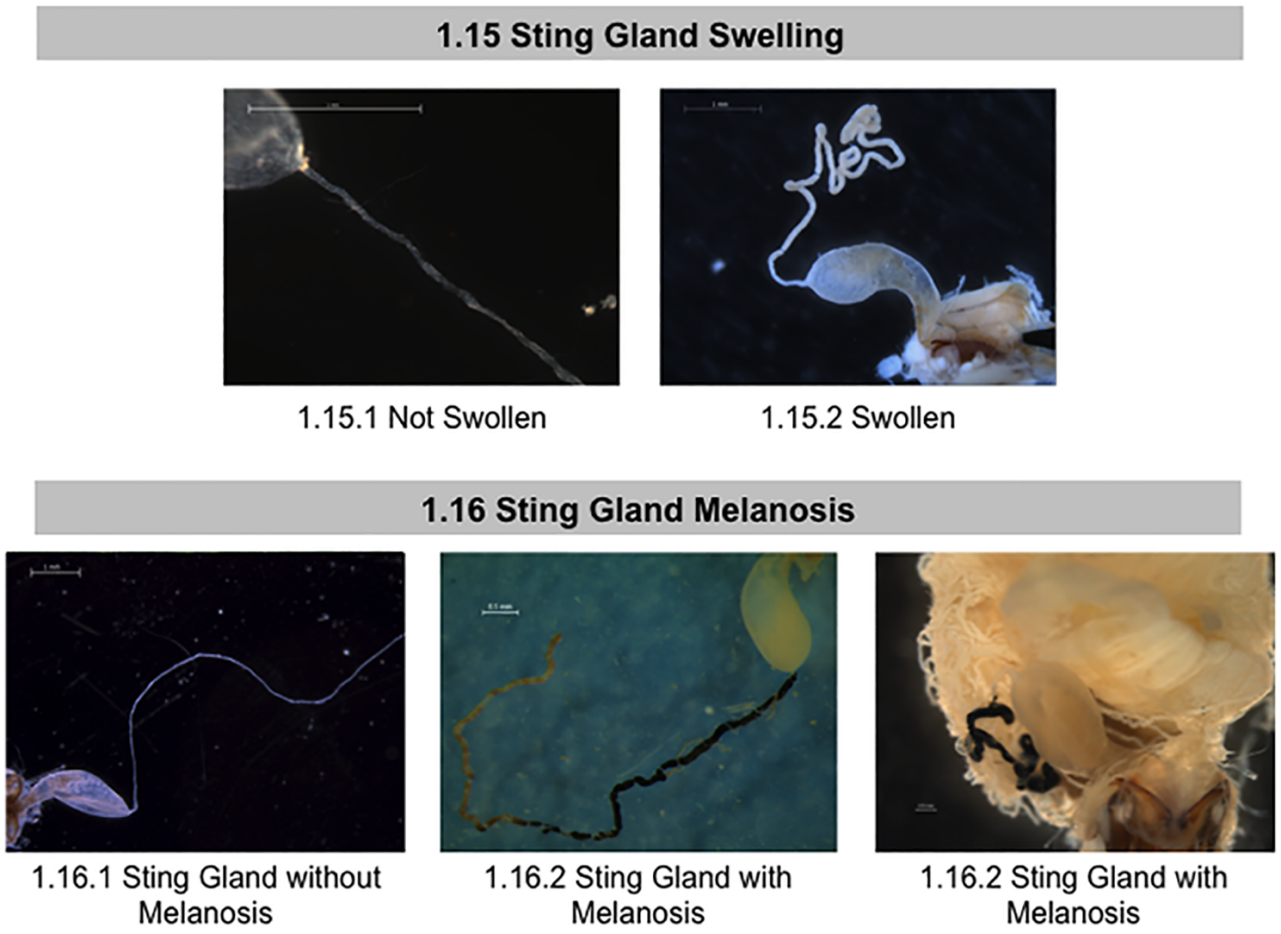
Normal and abnormal observed tissues in the sting glands of healthy bees and bees taken from CCD colonies. Location of the tissues and symptoms are described in the methods. These symptoms were used to the standardized scoring listed in Table 1. 1.15 Sting Gland Swelling; 1.16 Sting Gland Melanosis.

Following Snodgrass [35]as a guide for normal anatomy in the honey bee abdomen, we carefully examined each bee’s abdominal cavity, gastro-intestinal tract, and sting region for gross lesions and other symptoms, and scored 17 different visible conditions as listed in Table 1 and shown in Figs 1–8. Dissected bees were individually scored for these 17 conditions. Due to limited funds and personnel, we confined our examinations to the aforementioned regions. However, we recognize that additional examination of important honey bee glands and fat body may add further insight which was beyond the scope of this current work.

**Table 1:** Scoring criteria used to evaluate honey bee pathologies in the abdominal region corresponding to the images in Figs 1–8.

Variable/ Characteristic	Categorization (score)	Description
Black tissue	Absent (0)	
	Present (1)	Spots of discolored muscle or connective tissue present in the abdominal cavity
White nodules	Absent (0)	
	Present (1)	Opaque white nodules can be found in or on abdominal tissues
Ventriculus size	Small (0)	0.5-1mm wide by 4-5mm long
	Medium (1)	1–1.5mm wide by 4.5–5.5 mm long
	Large (2)	1.5–2.5mm wide by 5.5–6.5mm long
Ventriculus coloration	Light (0)	Sheath is > 2/3 white, cream-colored, or translucent
	Medium (1)	Sheath mostly tan to brown
	Dark (2)	Sheath almost entirely brown or black
Pyloric scarring	Absent (0)	
	Present (1)	Dark band (scar) running partially or entirely around the perimeter of the pylorus region
Malpighian tubule color	Clear (0)	≥ 2/3 of the tubules translucent to cream colored
	Slight discoloration (1)	≥ 2/3 of the tubules tan to brown
	Severe discoloration (2)	≥ 2/3 of the tubules brown to black
Malpighian tubule quantity	Normal (0)	≥ ~50 tubules present
	Reduced (1)	< ~50 tubules present
Malpighian tubule iridescence	Normal (0)	
	Iridescent spots present (1)	Small iridescent spots seen along the Malpighian tubules
Fecal matter color	Light (0)	White or very light yellow
	Medium (1)	Orange, deep yellow, red, or light brown
	Dark (2)	Dark brown
Rectum distension	¼ full (0)	Fills 0–33% of the abdominal cavity
	½ full (1)	Fills 33–66% of the abdominal cavity
	full (2)	Fills 66–100% of the abdominal cavity
Fecal matter consistency	Soft (0)	Ejects readily like a thick liquid from the rectum sac when probed
	Semi-hard (1)	Breaks apart in clumps when probed
	Hard (2)	Remains completely solid when probed
Enteroliths in rectum	Absent (0)	
	Present (1)	Hard concretions that look like small grains of rice present
Venom sac color	Translucent (0)	
	Discolored (1)	Any amount of discoloration visible
Venom sac debris	Absent (0)	
	Present (1)	Any sort of solid debris is present in the venom sac
Sting gland swelling	Normal (0)	Sting gland is of normal size
	Intermediate (1)	Sting gland is slightly larger than normal, and appears to have a second layer
	Very swollen (2)	Sting gland is extremely oversized
Sting gland tissue melanosis	Absent (0)	
	Present (1)	Sting gland contains areas of melanin
Sting gland color	Light (0)	Translucent to tan
	Medium (1)	Tan to brown
	Dark (2)	Brown to dark brown

We first examined all exposed abdominal tissue to detect black or melanized tissue and white nodules (Fig 1). These pathologies are not tissue specific, as melanization of tissues is the result of phenol oxidase activation in tissue and indicative of activation of the humoral immune responses [38]. We found white nodules present inside the abdominal cavity (Fig 1, panel 1.2.1), attached (Fig 1, panel 1.2.2) or embedded (Fig 1, panel 1.2.3) in tissue in the bees kept in 70% ethanol; these nodules are not apparent in freshly killed bees, indicating that tissue dehydration is needed for visualization[39]. These nodules are reported to consist primarily of nodules of hemocytes rich in the amino acid tyrosine [39]and can form in response to bacterial infection[40]. Similar nodules were previously found in pupae and adult bees parasitized by *Varroa*, implying that they are an immunological response to parasitic feeding[39].

We next examined the ventriculus (midgut) by measuring ventriculus color and size (Fig 2). The ventriculus is located between the posterior end of the proventriculus (a valve at the posterior portion of the foregut) and the anterior end of the proctodeum (hindgut), where the Malpighian tubules connect. The ventriculus is the principal site of digestion and absorption and is where the microsporidian parasite *Nosema* germinates[34]. Ventriculus color is dictated by the color of the surrounding muscle fibers and the outer connective tissue sheath[41,42]. In healthy bees, the ventriculus, when removed in saline, is translucent with reddish-brown appearance that may turn milky white or chalky upon *Nosema* infection[43]; although, careful research has demonstrated that this is not always the case in worker bees infected with either *Nosema apis* or *Nosema ceranae* [44–46]. When examining bees stored in alcohol, Snodgrass describes the healthy ventriculus as having an opaque white appearance[35]. We observed that after extended storage in alcohol or under poor storage conditions with high temperature, many of the bee’s tissues darken; to ensure that we accounted for natural darkening over time in storage, we scored each bee’s ventriculus color as light, medium, or dark relative to the color of the connective tissue and muscle (sheath) that is light or white in color in healthy newly autopsied bee. We also measured ventriculus size, which is dependent, in part, on how much the bee recently ate and/or the degree of *Nosema* infection [47].

Examination of the proctodeum included the pylorus region of the anterior intestine and the posterior intestine (rectum). The pyloric valve regulates passage from the ventriculus to the anterior intestine [34]. The anterior intestine is involved in ion regulation [48]and the rectum stores waste matter, while rectal pads in the rectum can absorb water from the feces[49]. We examined the pyloric valve for the presence or absence of a dark banded “scar” (Fig 3, red arrows). This symptom was first described in 1946 [50]and is a melanization response, which can be triggered by colonization by the gammaproteobacterium *Frischella perrara* [51]. The scarring is apparent even when looking through the outer layer of circular muscle fibers that surround this valve and is thus not due to tissue manipulation during dissection. The scar is typically 100–400 microns wide and partially or completely encircles the pyloric valve.

The Malpighian tubules (located at the anterior end of the intestine) open into the pyloric lumen immediately behind the pyloric valve region[35]. These are the principal organs of excretion and osmoregulation in insects, analogous to vertebrate kidneys [34,52]. In healthy honey bee adults, Malpighian tubules number 100 or more and are long, slender, translucent tubes [34]. Diseases such as *Malpighamoeba mellificae* [53]can cause tubule damage. We scored the Malpighian tubules for variations in overall color (Fig 3), number (Fig 4), and the presence or absence of iridescence (Fig 4, black arrows). Iridescent spots, also called “concretions” and “mineralized granules,” are dense bodies found within the Malpighian tubule epithelium or lumen[54]. These concretions often appear iridescent and result from mineral storage, xenobiotic detoxification, or an encapsulating bacterial immune response [52,55,56].

The remainder of the anterior intestine plays a crucial role in the passage of waste into the rectum[34]; however, we did not score this area as preliminary work revealed no obvious pathologies in this region.

Rectal fecal matter includes pollen exines, pollen lipid globules, and the sloughed epithelial cells from the ventriculus[34]. The rectum also contains a large community of presumably symbiotic microorganisms that may help decompose and detoxify undigested food [57,58]. These organisms may also provide benefit to their host by conferring immunity to some common bee diseases. Pesticides and veterinary honey bee medicines can influence the composition of honey bee microflora[59]. For instance, *Lactobacillus* resident in the hindgut may protect their host against *Paenibacillus larvae*, the causal agent of American foulbrood[60][61]. The rectum reabsorbs water from waste [62]and stores the waste until honey bees can take a cleansing flight[63]. If bees are unable to empty their rectums for extended periods of time, indigestible food components such as starch may ferment, enabling a proliferation of bacteria, yeasts, and microfungi, which can result in obvious signs of dysentery on frames and at the hive entrance [34]. The rectum’s ability to contract and distend allows for extensive fecal accumulation during winter and inclement weather[34].

For the purposes of this study, several components of the bees’ rectum and fecal matter were examined and scored: color, rectum size (which depends on fecal amount accumulated), content consistency, and the presence or absence of stone-like “enteroliths” (Figs 5 and 6). We use the term enteroliths, since these are hard, rounded and similar to the structures (enteroliths) described in the rectums of honey bee queens [64]or the mineral concretions called enteroliths that are sometimes found in the intestines of humans and horses[65]. In our studies, the contents of the rectum were visible through its outer layers of muscles. We quantified fecal matter accumulation by estimating the percentage of the abdomen a given rectum filled the abdomen; large 75–100% full, medium 30–75% full, and small 0–30% full (Fig 5, panel 1.10). To measure fecal matter consistency, we used forceps to break through the rectum and probed the fecal matter held inside. When probing, we noted if the contents were hard, semi-soft or very soft (Fig 6, panel 1.11). We also noted the presence/absence of enteroliths that looked like small grains of rice contained in the rectum (Fig 6, panel 1.12).

Next, we examined the sting apparatus for abnormal pathologies. The sting of *Apis mellifera* is contained within the abdomen in segment VII, and consists of the venom gland (also called poison or acid gland) and a sting gland. The venom gland consists of a sac that opens by a thin neck into the base of a bulbous cavity near the stinger[35]. The sting gland is long and strand-like. Investigation began with the venom sac, noting variations in color (Fig 7, panel 1.13). We assessed the sting gland for discoloration, swelling, and the presence of the black-pigment melanin (melanosis) (Fig 8, panels 1.14–1.16). The color of sting glands ranged from translucent to tan to dark brown, with either solid or patchy melanization.

### Comparison of bees from CCD vs. control apiaries

Colonies in an apiary with CCD-symptomatic hives may not have started to collapse but may already have been suffering from ill health without yet expressing symptoms. For this reason, we compared all bees collected from colonies in apiaries suffering from CCD with bees collected from colonies in apiaries with no sign of the disorder (Table 2). Seven pathologies showed different disease manifestation patterns in apiaries with and without CCD: Malpighian tubule iridescence, rectum distension, fecal matter consistency, rectal enteroliths, venom sac color, sting gland swelling, and sting-gland melanosis. CCD apiaries were more likely to have bees with rectums that were less than half-full as compared to bees that came from non-CCD apiaries. The content of the rectums of bees from CCD apiaries also was softer. Bees collected from CCD apiaries were 7.7 times more likely to have rectal enteroliths when compared to bees collected from control apiaries. Bees from CCD apiaries were more likely to have sting gland tissue melanosis. In contrast, bees from a CCD apiary were less likely to have discolored venom sacs and swollen sting glands than bees from control apiaries.

**Table 2:** Frequency of symptoms in bees collected from CCD *versus* non-CCD apiaries, as colonies may already be in decline from CCD without visibly recognizable symptoms. Results shown as the percent of individual bees with each score (prevalence). Test results are either from likelihood ratio tests or, if marked with “§”, chi-squared tests. “*” indicates statistical significance at α = 0.05.

	Bees from CCD apiary				Bees from non-CCD apiary				Likelihood ratio test (CCD *vs*. non-CCD)	
Variable	N	Score 0 (%)	Score 1 (%)	Score 2 (%)	N	Score 0(%)	Score 1(%)	Score 2(%)	χ^2^	P
Black tissue	525	53.3	46.7	NA	180	70.0	30.0	NA	1.79	0.18
White nodules	531	94.7	5.3	NA	180	91.7	8.3	NA	0.007	0.93
Ventriculus size	501	31.5	49.7	18.8	168	31.0	53.0	16.1	0.16	0.69
Ventriculus coloration	515	28.5	59.8	11.7	174	25.9	59.2	14.9	0.36	0.55
Pyloric scarring	515	88.7	11.3	NA	176	89.8	10.2	NA	0.48	0.49
Malpighian tubule color	515	6.4	74.4	19.2	177	5.6	74.0	20.3	0.13	0.72
Malpighian tubule quantity	513	54.8	45.2	NA	174	67.2	32.8	NA	1.30	0.25
Malpighian tubule iridescence	529	83.4	16.6	NA	180	97.8	2.2	NA	23.45§	<0.0001*
Fecal matter color	527	3.2	87.7	9.1	178	2.2	97.2	0.6	1.59	0.21
Rectum distension	529	26.3	53.1	20.6	178	11.8	34.3	53.9	23.01	<0.0001*
Fecal matter consistency	525	67.0	25.7	7.2	178	52.8	23.6	23.6	5.78	0.02*
Rectal stones	357	95.0	5.0	NA	150	99.3	0.7	NA	6.35	0.01*
Venom sac color	495	41.6	58.4	NA	178	19.7	80.3	NA	7.29	0.007*
Sting gland swelling	475	25.3	74.7	NA	177	13.0	87.0	NA	10.63	0.001*
Sting gland tissue melanosis	476	54.0	46.0	NA	176	79.0	21.0	NA	6.07	0.01*
Sting gland color	476	19.5	80.5	NA	177	15.8	84.2	NA	0.31	0.58

**Fig 9:**
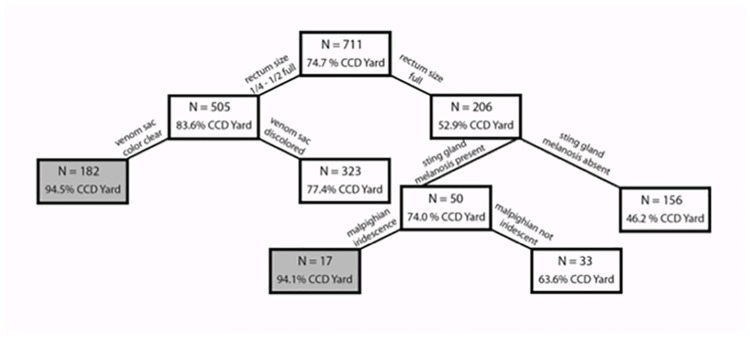
Classification tree of the factors distinguishing apiaries with CCD symptoms (n = 531) from control apiaries without any CCD symptomatic colonies (n = 180). Boxes shaded in gray indicate a terminal node, where the decision tree ends. This classification tree had a specificity of 81.1% (95% CI: 74.6–86.5) (i.e., the ability to correctly identify non-CCD apiaries) and a sensitivity of 70.8% (95% CI: 66.7–74.6) (i.e., the ability to correctly identify CCD apiaries).

CART analysis highlighted four of the physio-pathological traits described above as having high predictive power (i.e. relative importance) for the presence of CCD in an apiary (Fig 9). The most informative with a power = 100 was rectum distension (size), followed by Malpighian tubule iridescence (power = 83.65), fecal matter consistency (power = 74.49), and venom sac color (power = 53.02).

### Comparison of bees from CCD symptomatic colonies vs. non-symptomatic colonies

We compared bees collected from colonies showing CCD symptoms with bees taken from non-symptomatic colonies. A subset of the physio-pathological traits (Malpighian tubule iridescence, rectum distension and the presence of rectal enteroliths) was differentially expressed in CCD and non-CCD colonies (Table 3). Iridescent spots in the Malpighian tubules were more prevalent in bees from CCD symptomatic colonies than in bees from non-symptomatic colonies. Compared to bees from colonies without CCD, bees from CCD colonies were likely to have rectums that were less than ½ full. Rectal enteroliths occurred more than 3 times as frequently in CCD suffering bees compared to non-CCD suffering bees. This subset of physio-pathological traits (rectal size and presence of enteroliths) was similarly differentially expressed in CCD positive apiaries and colonies.

**Table 3:** Frequency of symptoms in bees collected from CCD symptomatic verses non-CCD colonies, shown as the percent of individual bees with each score (prevalence). Test results are either from likelihood ratio tests or, if marked with “§”, chi-squared tests. “*” indicates statistical significance at α = 0.05.

	Bees from CCD Symptomatic colony				Bees from non-symptomatic colony				CCD vs. non-CCD	
Variable	N	Score 0 (%)	Score 1 (%)	Score 2 (%)	N	Score 0 (%)	Score 1 (%)	Score 2 (%)	χ^2^	P
Black tissue	346	57.5	42.4	NA	359	57.7	42.3	NA	0.005	0.95
White nodules	350	95.7	4.3	NA	361	92.2	7.8	NA	0.001	0.97
Ventriculus size	327	30.9	47.1	22.0	342	31.9	53.8	14.3	0.67	0.41
Ventriculus coloration	339	31.0	57.8	11.2	350	24.9	61.4	13.7	0.87	0.35
Pyloric scarring	341	90.3	9.7	NA	350	87.7	12.3	NA	0.17	0.68
Malpighian tubule color	338	7.4	72.8	19.8	354	5.1	75.7	19.2	0.13	0.72
Malpighian tubule quantity	336	55.4	44.6	NA	351	60.4	39.6	NA	0.15	0.69
Malpighian tubule iridescence	349	82.5	17.5	NA	360	91.4	8.6	NA	11.57§	0.0007*
Fecal matter color	346	1.7	89.3	9.0	359	4.2	90.8	5.0	1.34	0.25
Rectum distension	348	24.1	56.0	19.8	359	21.2	40.9	37.9	5.65	0.02*
Fecal matter consistency	344	65.4	27.6	7.0	359	61.6	22.8	15.6	1.93	0.17
Rectal Stones	236	94.1	5.9	NA	271	98.2	1.8	NA	4.94	0.03*
Venom sac color	326	39.6	60.4	NA	347	32.3	67.7	NA	0.77	0.38
Sting gland swelling	315	24.8	75.2	NA	337	19.3	80.7	NA	2.54	0.11
Sting gland tissue melanosis	316	57.0	43.0	NA	336	64.3	35.7	NA	0.45	0.50
Sting gland color	316	21.5	78.5	NA	337	15.7	84.3	NA	0.01	0.92

CART analysis revealed that these four characteristics continued to strongly influence the decision tree when discriminating between symptomatic and non-symptomatic colonies (S1 Fig), but the order of importance shifted when compared to the CART analysis that distinguished between CCD apiaries and non-CCD apiaries (see Fig 9). Rectal fecal-matter consistency had the greatest relative importance (power = 100), followed closely by rectum size (98.84). An additional 8 factors contributed to the most parsimonious decision tree, including sting gland color (66.96), venom sac color (58.72), sting gland melanosis (54.95) Malpighian tubule iridescence (54.51), rectal enteroliths (51.00), rectum color (50.74) and Malpighian tubule color (50.65). The resulting decision tree had a specificity of 59.3% (95% CI: 54.0–64.4) and sensitivity of 60.3% (95% CI: 54.9–65.4). The lower overall tree specificity and sensitivity indicated that one can correctly distinguish between CCD and control apiaries with greater certainty than one can distinguish between CCD and non-CCD colonies that may or may not share the same apiary.

### Effects of age on physio-pathological traits

**Fig 10:**
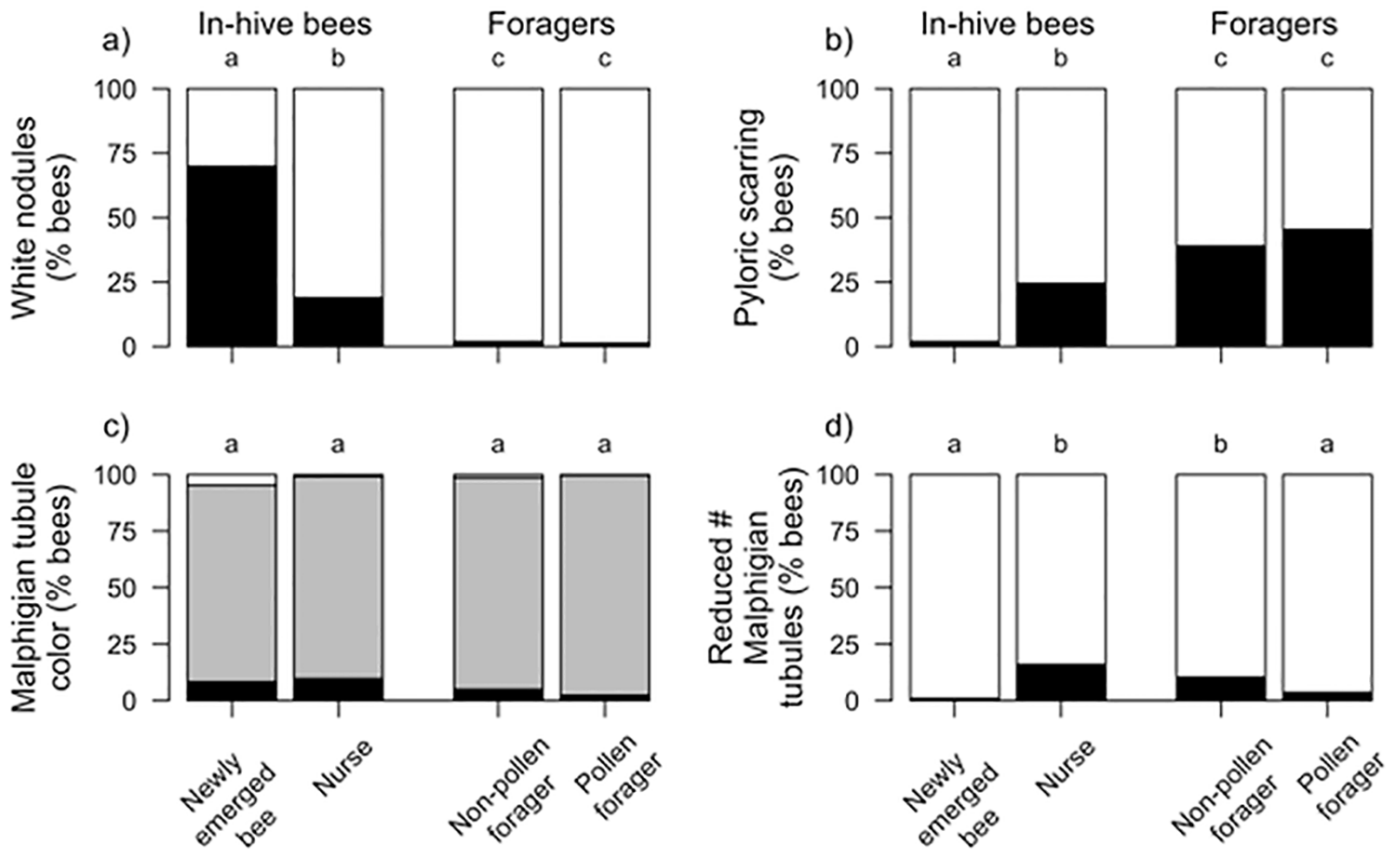
Relative frequency of four physio-pathological traits that varied among age cohorts. A) White Nodules (Black = presence, White = absence), B) Pyloric scarring (Black = presence, White = absence), C) Malpighian Tubule Color (Black = severe discoloration, Gray = slight discoloration, White = no discoloration), D) Malpighian tubule number (Black = reduced number, White = normal number). Significant differences among cohorts for each trait is indicated by different letters (alpha = 0.05, Tukey HSD).

**Fig 11:**
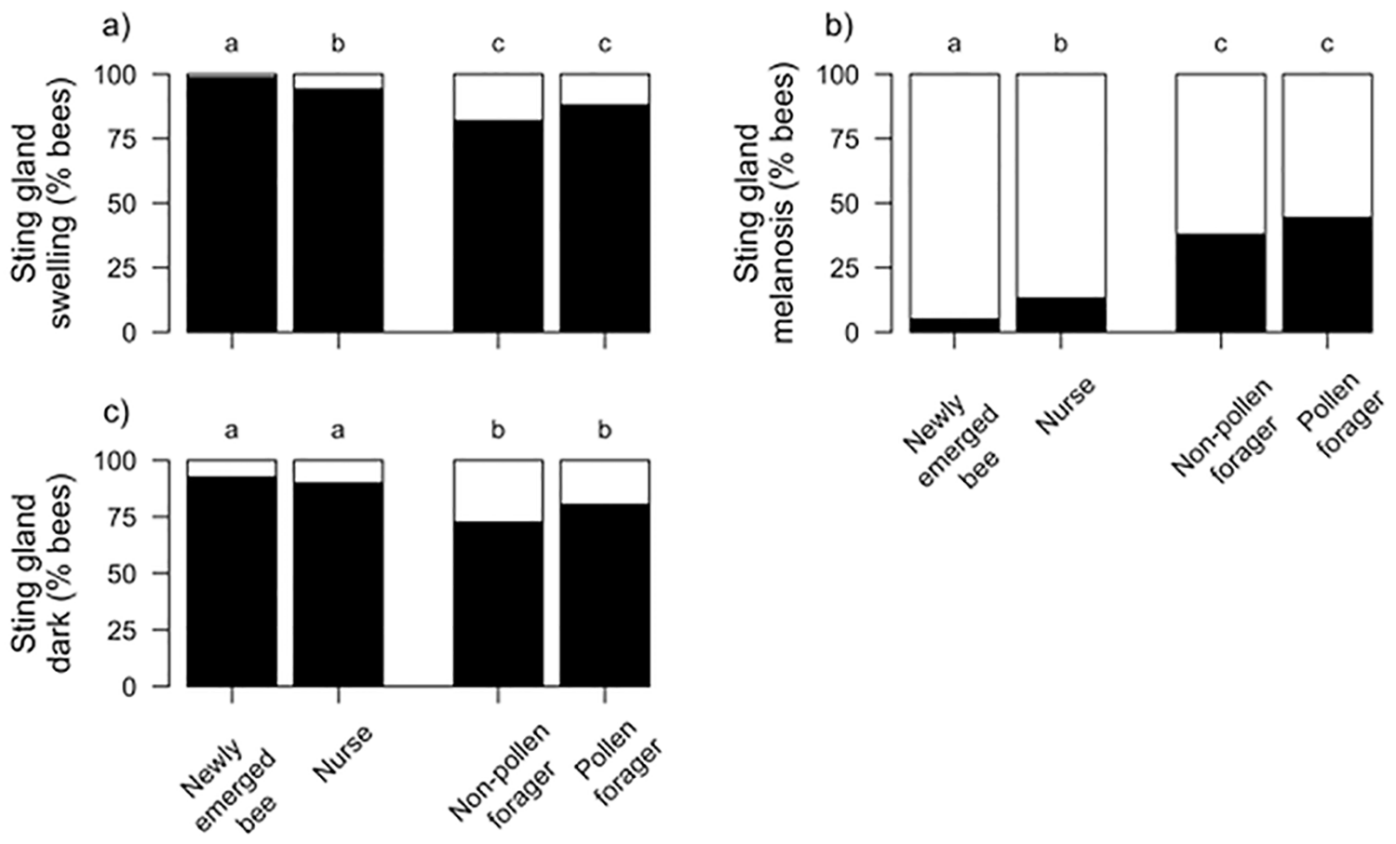
Relative frequency of pathologies associated with the sting gland that varied among age cohorts. A) Sting gland swelling (Black = presence, White = absence), B) Sting gland tissue melanosis (Black = presence, White = absence), C) Sting gland color (Black = presence, White = absence). Significant differences (α = 0.05, Tukey HSD) indicated by different letters.

Adult bee task is closely associated with age, with newly-emerged bees maturing into nurses and then transitioning to foraging. We compared pathologies across four age-related tasks (newly emerged, nurse, non-pollen forager, and pollen forager) and found significant differences among six of the seven physio-pathological traits we investigated (Figs 10 and 11): white nodules (χ^2^ = 552.9, df = 3, *p* < 0.0001), pyloric scarring (χ^2^ = 213.6, df = 3, *p* < 0.0001), Malpighian tubule quantity (χ^2^ = 70.1, df = 3, *p* < 0.0001), sting gland swelling (χ^2^ = 63.3, df = 3, *p* < 0.0001), sting gland tissue melanosis (χ^2^ = 193.2, df = 3, *p* < 0.0001), and sting gland color (χ^2^ = 56.4, df = 3, *p* < 0.0001). Malpighian tubule color did not differ between bees of different ages (χ^2^ = 0.8, df = 3, *p* = 0.84). The prevalence of white nodules and sting-gland swelling declined with age. White nodules were found more often in newly-emerged workers than in any other age group. Sting gland discoloration also declined with age but was equally prevalent in newly-emerged workers and nurse bees. Pyloric scarring and melanized sting glands had the opposite pattern, increasing in prevalence with age. Malpighian tubule quantity was significantly reduced in newly-emerged workers as compared to all other worker age classes. Pathology frequencies were always equally distributed between pollen and non-pollen foragers.

We further classified the cohorts of healthy bees into in-hive bees (young) and foragers (old), to determine if physio-pathological traits observed in CCD colonies were a factor of the bees being young instead of ill. Bees left in CCD colonies are assumed to be young, given the presence of developing brood and newly-emerged bees, but the exact age or age-related task is undeterminable given the highly-reduced worker-bee populations. The transition in healthy bees between in-hive nursing and outside foraging is accompanied by a suite of physiological changes [16,66,67]and these may be reflected in changes in internal tissues. Five of the above physio-pathological traits differed significantly between in-hive bees and foragers. Younger bees were more associated with these: white nodules (χ^2^ = 376.3, df = 1, *p* < 0.0001), pyloric scarring (χ^2^ = 125.2, df = 1, *p* < 0.0001), sting gland swelling (χ^2^ = 48.7, df = 1, *p* < 0.0001), sting gland tissue melanosis (χ^2^ = 175.8, df = 1, *p* < 0.0001), and sting gland color (χ^2^ = 50.2, df = 1, *p* < 0.0001). Malpighian tubule quantity, which was significantly reduced in newly-emerged bees, did not differ when we grouped bees by in-hive bees vs. forager bees (χ^2^ = 2.12, df = 1, *p* = 0.15). Malpighian tubule color was also independent of this transition between in-hive tasks and foraging (χ^2^ = 0.4, df = 1, *p* = 0.53).

**Fig 12:**
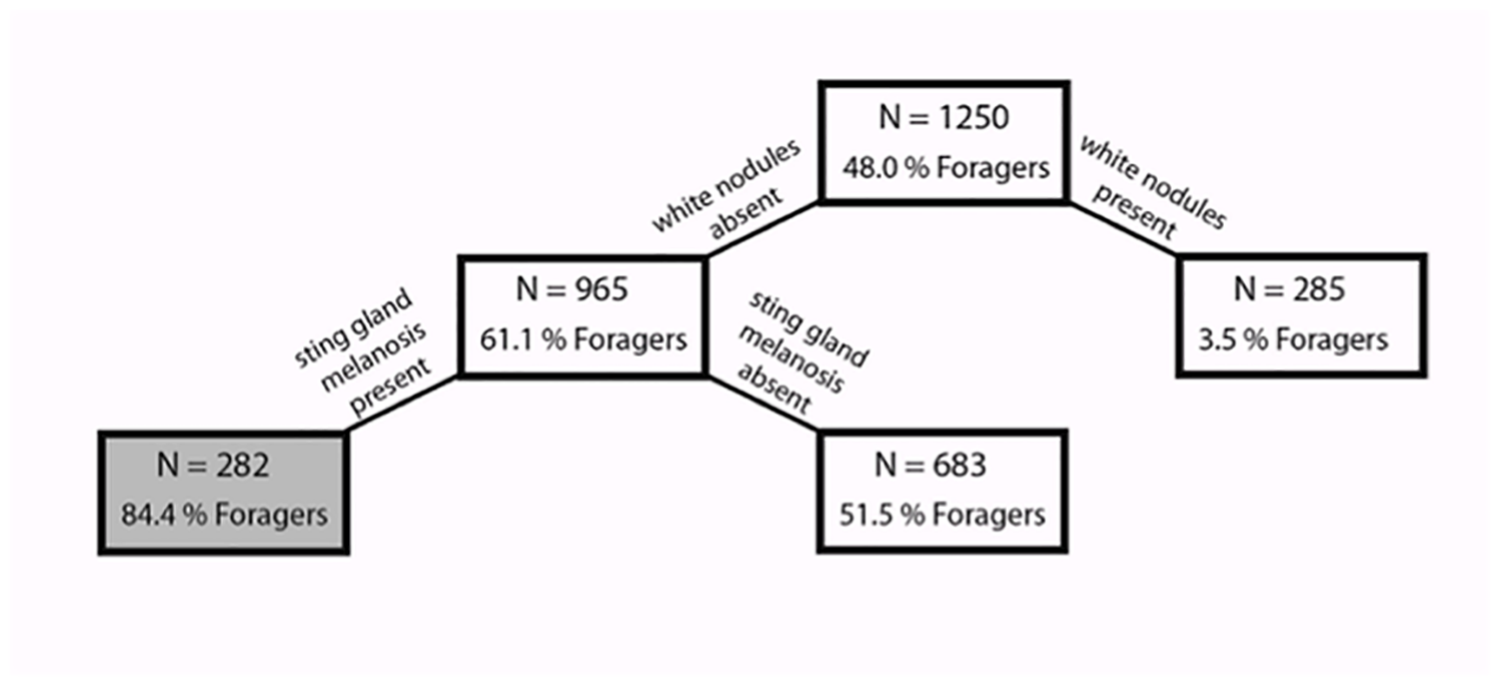
Classification tree of the factors distinguishing bee age for foragers (n = 600) from in-hive bees (n = 650 with newly-emerged and nurses pooled together). Boxes shaded in gray indicate a terminal node, where the decision tree ends.

CART analysis revealed that white nodules had the greatest predictive power to distinguish between in-hive bees and foragers (power = 100), while sting gland melanosis also contributed (power = 28.58). Consistent with regressions, our classification tree found that in-hive bees were more likely to have white nodules and less likely to show sting gland melanosis (Fig 12). Overall the tree had 72.2% (95% CI: 68.4–75.7) specificity and 82.0% (95% CI: 78.8–84.9) sensitivity. CART analysis could not distinguish between different age-associated tasks (i.e., distinguish between newly-emerged workers and in-hive nurse bees or between non-pollen foragers pollen foragers).

## Discussion

Our results demonstrate that physio-pathological traits associated with collapsing colonies are present even before all colonies in an apiary become symptomatic, suggesting that studying bees from apparently healthy colonies can be used to detect diseased colonies prior to collapse. A set of physical characteristics (rectum distension, Malpighian tubule iridescence, fecal matter consistency, and venom sac color (Figs 5–7)) can identify CCD prior to colony demise. Comparing symptoms between bees from CCD apiaries and non-CCD apiaries revealed that CCD apiaries tended to have partially filled rectums, soft fecal matter, rectal enteroliths and sting gland melanosis. Sting gland melanosis was associated more often with older foragers and not young bees, so it was surprising to see it more frequently in bees from CCD apiaries than non-CCD apiaries, given that it is assumed that bees in CCD apiaries are recently emerged bees.

Using these physio-pathological traits to distinguish between bees from CCD-symptomatic versus non-symptomatic colonies was more difficult, since the decision trees resulting from CART analysis had significantly lower sensitivity and specificity. The rectum’s characteristics played an important role in this decision tree, with fecal matter consistency and rectal distension (size) contributing the greatest power. Regressions revealed that Malpighian tubule iridescence was significantly more common in symptomatic bees, as were rectal enteroliths.

Physio-pathological traits associated with CCD were not the result of the collapsed colonies containing only young bees. The majority of physio-pathological traits that differed among bee castes (white nodules, pyloric scarring, Malpighian tubule quantity, and sting gland color) were not the same as those associated with CCD colonies. In fact, younger bees consistently had a very different appearance of their internal tissues as compared to those of bees from CCD-symptomatic apiaries and colonies. For example, the sting glands in bees in CCD-symptomatic colonies and apiaries were more likely to be melanized, while younger in-hive bees were less likely to show sting gland melanization, suggesting an active immune response was occurring in this tissue in the CCD bees. Not all characteristics that were examined in the CCD analysis were investigated in the age study, since preliminary dissections across age groups revealed no changes in the rectal characteristics and rectal enteroliths were never detected in the bees from healthy colonies. Now that pathological symptoms tied to CCD are identified, the mechanisms that underlie these physio-pathological traits beg investigation.

The importance of the size and contents of the rectum when distinguishing between both CCD and control apiaries and between symptomatic and non-symptomatic colonies suggests possible nutritional or water-balance disruptions, as the rectum serves as a nutritional waste storage vessel during confinement. Our results suggest CCD bees may be unable to either produce or retain fecal waste in the rectum during extended periods of confinement, such as over winter or during rainy weather. Bees in both apiaries and colonies having CCD symptoms had significantly less fecal matter in the rectum. There was no reason to expect that these bees would retain fecal waste since bees had been in located in Southern states for several weeks prior to collapse and should have been taking cleansing flights in warm weather. It is more likely that bees from CCD colonies may have been underfed, especially with pollen, resulting in less fecal matter than was found in healthy bees (less food = less feces). Rectal enteroliths, which were more common in bees from CCD-positive apiaries and almost never detected in healthy bees, may indicate a disrupted excretory physiology. Rectal enteroliths have previously been reported in queens, where they were associated with egg-laying cessation, abnormal eggs, and swollen rectums. In the 1960’s, the researcher Fyg performed chemical analyses and found uric acid and minerals in the enterolith concretions in queens; he hypothesized these were indicative of constipation and poor osmoregulation [64,68].

Malpighian tubule iridescence was more common in bees from CCD apiaries and symptomatic colonies. This observed pathophysiology may have several causes, including exposure to pathogens and/or different environmental toxins[52,69,70]. In wild-type *Drosophila*, exposure to environmental toxins such as ethylene glycol or melamine can result in concretions that are iridescent[70]. These concretions indicate a disruption in osmoregulation via the ion and organic solute transporters[52,70]. Besides involvement in osmoregulation, Malpighian tubules also have important roles in detoxification with an expression of P450 genes [52]used for effective xenobiotic metabolism[71]. The Malpighian tubules can also mount an immune response independent of the fat body[72]. No pathogen has a known association with iridescent Malpighian tubules; however, this needs further exploration. The Malpighian tubules play a variety of protective roles [73]in addition to excretion, and their impairment could help contribute to increased colony losses in the face of environmental stressors.

White nodules observed in the hemocoel were common to newly-emerged and nurse bees. CART analysis demonstrated that these white nodules had the greatest predictive power when distinguishing between in-hive bees and foragers. Nodulation/encapsulation is a highly conserved physiological response that entraps micro-organisms through the aggregation of hemocytes, a key component of the insect cellular immune system [39,74]. The presence of the nodules suggests a highly active immune system. Given that the nodules were common to newly-emerged bees, they may be a remnant from tissue remodeling that occurs during metamorphosis and pupation [75]. In *Drosophila* and stingless bees, the larval fat body disassociates into single cells; some of the cells persist into the newly-emerged adult and then undergo cell death [75,76]. It is possible that this process is slower in honey bees, with the majority of dying cells being recognized and engulfed in hemocyte aggregations in newly-emerged bees. Some white nodules were still present in nurse bees. Alternatively, the nodulation may result from hemocyte aggregation in response to parasitization by varroa mites, which feed predominantly on developing pupae and damage may be apparent in newly-emerged bees.

The pathology of the rectum and Malpighian tubules of CCD bees suggests that osmoregulation and/or ion balance are key processes worthy of further investigation. These disorders may indicate that stresses are affecting osmoregulation and ion balance, both at an individual and colony level [77]. As brood rearing and colony homeostasis require constant temperatures regulated via water evaporation [78], individual workers suffering from poor osmoregulation could leave the colony susceptible to temperature fluctuations and desiccation with detrimental effects on the quality of brood rearing, food storage, and colony survival. Malpighian tubule disturbances have been linked to environmental toxins in *Drosophila* [70], and the role of xenobiotics in the development of Malpighian tubule pathologies in honey bees needs further investigation.

Our data clearly demonstrate that pathophysiology is a useful tool in evaluating colony health prior to demise; and that particular pathologies will likely serve as biomarkers for diagnosis or prediction of colony health. Additional research is needed to determine if these physio-pathological traits are seen in other colony losses beyond those with CCD symptoms. The addition of our necropsy methods to the toolbox used by researchers and beekeepers will be valuable in evaluating colony health and in predicting the future of their bees.

## Methods

### Selecting CCD and “healthy” colonies

Colonies used were sampled in 2007 as part of an epidemiological study that sought to determine potential causes of CCD[20]; we collected bees from 13 apiaries located in either Florida or California during January and February 2007. Colonies were classified in the field as coming from apiaries lacking CCD (18 control colonies from “non-CCD apiaries”) or apiaries having colonies with CCD (55 colonies from “CCD apiaries”). Out of the 55 colonies in CCD apiaries, 36 were in apparent decline (“symptomatic CCD”), while 19 appeared healthy (“non-symptomatic CCD”) at the time of collection. For CCD colonies, we examined approximately 10 bees from each colony, with samples taken randomly from bees collected from the brood nest (when brood was present) [20].

For healthy colonies, these were from one of the beekeeping operations originally sampled; the colonies were transferred to Pennsylvania and kept in one of the author’s backyards in 2008. The colonies were observed for several months to verify health. From these colonies, bees from various age groups were sampled, with approximately 50 bees of each group taken from each of six healthy colonies. All of the bee samples were initially frozen at -80°C and then stored in 70% ethanol at room temperature.

### Bee dissections and gross lesion standardized scoring

Bees necropsied (usually 10 at a time) were removed from their storage bottles and allowed to dry slightly on a paper towel. We made two shallow incisions along the lateral sides of a bee’s abdomen, in the pleural region, using forceps and micro-scissors (EMS high-precision tweezers, style 5B with extra fine and bent tips; straight Dumont positive-action tweezers, Style SS; Vannas Capsulotomy scissors with 5 mm straight blade). Incisions started at the posterior end of the abdomen and ended near the petiole. Unless otherwise noted, once the incisions were made, we detached the abdomen from the thorax and placed it in a Petri-dish containing 70% ethanol to facilitate tissue separation. Under a stereo microscope (Leica MZ16 A), we removed abdominal sclerites, clearing away the sternites and tergites from around the gastro-intestinal tract until the internal tissues were completely visible. Following Snodgrass[35], we carefully examined each bee’s abdominal cavity, gastrointestinal tract, and sting region for gross lesions and other symptoms, and scored 17 different visible conditions as listed in Table 1.

### Effects of bee age and task on gross lesions and symptoms

To determine whether pathologies seen at greater frequency in bees from CCD colonies (assumed to contain primarily young bees performing in-nest tasks) are due to CCD or are the result of skewed cohort composition of the colony, we performed autopsies on healthy worker bees taken from healthy colonies that were performing distinctive tasks that link to age. We chose six CCD-negative *Apis mellifera* colonies for cohort-specific sampling. From each of these colonies we sampled and dissected 50 bees from each of the following cohorts: newly-emerged workers (N.E.W.), nurses, pollen foragers, and non-pollen foragers (carrying nectar, water, or returning empty). Bees transition among these cohorts following an age-dependent polyethism, with bees from healthy colonies transitioning from newly-emerged adult bees to nurses, then to foraging. Newly-emerged workers are distinguished by their fluffy thorax hairs, which remain distinctive for a short time after emergence. These bees were removed from frames with visible emerging bees. Nurse bees feed larvae and the queen and are found in the interior portions of the nest actively engaged in attending the brood. We collected foragers by placing a screen over the hive entrance and sampled bees attempting to re-enter the nest. Bees carrying pollen on their corbiculae were deemed pollen foragers, while those without pollen loads were labeled as non-pollen foragers. Necropsy protocols followed those described above. We did not color mark bees and allow them to age into their respective tasks, because we wanted minimal disturbance to the colony. We sought to capture the normal age distribution for each of these worker task roles as the comparative bees sampled from CCD colonies were of unknown, but presumably mixed ages. These bees were frozen and immersed in 70% ethanol in the same manner as the bees taken from CCD colonies. We limited our investigations to seven physio-pathological traits: white nodules, pyloric scarring, Malpighian tubule color, Malpighian tubule quantity, sting gland swelling, sting gland tissue melanosis, and sting gland color. Based upon preliminary studies with more than 50 healthy bees, we did not examine the rectum, given that all bees had full rectums containing soft contents and no enteroliths.

### Data analysis

Statistical analyses were carried out in R[79]. We first assessed whether each individual gross lesion or pathology predicted CCD status. We compared the pathologies diagnosed in bees as either coming or not coming from CCD-effected colonies or apiaries using generalized linear mixed models (GLMMs) with pathology scores as the response variable, the apiary’s or colony’s CCD status as the fixed effect and the bee’s colony as a random effect. The apiary-level analyses permit detection (if present) of CCD-related traits when colonies are suffering from CCD without yet expressing symptoms of collapse. Colonies categorized as non-symptomatic included those from both CCD and control apiaries. Binary physio-pathological traits were analyzed via logistic regressions with the lme4 R package [80], and ordinal pathologies via cumulative logistic regressions with the ordinal R package[81].

When CCD status significantly affected pathology scores, we calculated odds ratios comparing pathology scores in colonies or apiaries with and without CCD. For two pathology types, Malpighian tubule iridescence and sting gland swelling, the pathology or its absence was sufficiently rare that GLMM results were too imprecise. For these, we employed chi-squared tests to assess whether pathology prevalence differed between bees from colonies or apiaries with and without CCD.

We analyzed the effects of bee age on gross lesions and pathologies using GLMMs similar to those described above. Here, the fixed effect was bee age rather than CCD status. We followed the common practice of analyzing the ordinal response variable (Malpighian tubule color) via Poisson regression to facilitate post-hoc testing within the same framework that we used for logistic regression. To determine significant differences between age class pairs, we employed Tukey HSD tests using the Multcomp R package [82].

To better understand how combinations of pathologies or gross lesions are related to CCD, we conducted classification and regression tree (CART) analyses. A CART analysis is a non-linear and non-parametric model that is fitted by binary recursive partitioning of multidimensional covariate space[83]. Using Salford Predictive Modeler software (Salford Systems, San Diego, CA, USA), the analysis successively splits the dataset into increasingly homogeneous subsets until it is stratified to meet specified criteria. The Gini index was used as the splitting method, and 10-fold cross-validation was used to test the predictive capacity of the obtained trees. CART performs cross validation by growing maximal trees on subsets of data then calculating error rates based on unused portions of the data set. To accomplish this, CART divides the data set into 10 randomly selected and roughly equal parts, with each “part” containing a similar distribution of data from the populations of interest (e.g., CCD apiaries and non-CCD apiaries). CART then uses the first 9 parts of the data, constructs the largest possible tree, and uses the remaining 1/10 of the data to obtain initial estimates of the error rate of the selected sub-tree. The process is repeated using different combinations of the remaining 9 subsets of data and a different 1/10 data sub-set to test the resulting tree. This process is repeated until each 1/10 sub-set of the data has been used to test a tree that was grown using a 9/10 data sub set. The results of the 10 mini-tests are then combined to calculate error rates for trees of each possible size; these error rates are applied to prune the tree grown using the entire data set. The consequence of this process is a set of fairly reliable estimates of the independent predictive accuracy of the tree, even when some of the data for independent variables are incomplete and/or comparatively small. Further details about CART are presented in previously published articles (e.g.,[84–87][88].

We constructed three classification trees using CART analysis. The first tree differentiated between CCD apiaries and non-CCD apiaries. The second tree distinguished CCD symptomatic bees from non-symptomatic bees. The final tree analyzed characteristics due to honey bee task, thus allowing determination of factors that change with bee age.

## Supporting information

S1 Fig CART analysis of bees from CCD symptomatic and non-symptomatic colonies from all investigated apiaries. (PDF)

S1 Data Set Autopsy data from individual bees from CCD and non-CCD colonies in CCD and non-CCD apiaries. (CSV)

S2 Data Set Autopsy data from cohorts of bees of different metamorphic stages, sexes, and age-related cohorts. (CSV)

## References

[1] Lee KV, Steinhauer N, Rennich K, Wilson ME, Tarpy DR, et al (2015) A national survey of managed honey bee 2013–2014 annual colony losses in the USA. Apidologie 46: 292–305.

[2] Spleen AM, Lengerich EJ, Rennich K, Caron D, Rose R, et al (2013) A national survey of managed honey bee 2011–12 winter colony losses in the United States: results from the Bee Informed Partnership. Journal of Apicultural Research 52: 44–53.

[3] Seitz N, Traynor KS, Steinhauer N, Rennich K, Wilson ME, et al (2016) A national survey of managed honey bee 2014–2015 annual colony losses in the USA. Journal of Apicultural Research: 1–12.

[4] Steinhauer NA, Rennich K, Wilson ME, Caron DM, Lengerich EJ, et al (2014) A national survey of managed honey bee 2012–2013 annual colony losses in the USA: results from the Bee Informed Partnership. Journal of Apicultural Research 53: 1–18.

[5] vanEngelsdorp D, Caron D, Hayes J, Underwood R, Henson M, et al (2012) A national survey of managed honey bee 2010–11 winter colony losses in the USA: results from the Bee Informed Partnership. Journal of Apicultural Research 51: 115–124.

[6] vanEngelsdorp D, Hayes J, Underwood R, Pettis J (2010) A survey of honey bee colony losses in the United States, fall 2008 to spring 2009. Journal of Apicultural Research 49: 7–14.

[7] vanEngelsdorp D, Hayes J, Underwood RM, Caron D, Pettis J (2011) A survey of managed honey bee colony losses in the USA, fall 2009 to winter 2010. Journal of Apicultural Research 50: 1–10.

[8] vanEngelsdorp D, Hayes J Jr., Underwood RM, Pettis J (2008) A Survey of Honey Bee Colony Losses in the U.S., Fall 2007 to Spring 2008. PLoS ONE 3: e4071 doi: 10.1371/journal.pone.0004071 19115015 10.1371/journal.pone.0004071 PMC2606032

[9] Johnson RM, Ellis MD, Mullin CA, Frazier M (2010) Pesticides and honey bee toxicity—USA. Apidologie 41: 312–331.

[10] Alaux C, Ducloz F, Crauser D, Le Conte Y (2010) Diet effects on honeybee immunocompetence. Biol Lett 6: 562–565. doi: 10.1098/rsbl.2009.0986 20089536 10.1098/rsbl.2009.0986 PMC2936196

[11] Francis RM, Nielsen SL, Kryger P (2013) Varroa-Virus Interaction in Collapsing Honey Bee Colonies. PLoS ONE 8. 10.1371/journal.pone.0057540 PMC3602523 23526946

[12] Rennich K, Pettis J, VanEngelsdorp D, Bozarth R, Eversole H, et al. (2012) 2011–2012 National Honey Bee Pests and Diseases Survey Report. USDA. 17 p.

[13] Staveley JP, Law SA, Fairbrother A, Menzie CA (2014) A Causal Analysis of Observed Declines in Managed Honey Bees (Apis mellifera). Human and Ecological Risk Assessment 20: 566–591. doi: 10.1080/10807039.2013.831263 24363549 10.1080/10807039.2013.831263 PMC3869053

[14] Barron AB (2015) Death of the bee hive: understanding the failure of an insect society. Current Opinion in Insect Science 10: 45–50. 10.1016/j.cois.2015.04.004 29588013

[15] Seeley TD (1995) The wisdom of the hive: the social physiology of honey bee colonies. Cambridge, Mass.: Harvard University Press xiv, 295 p. p.

[16] Page RE, Robinson GE (1991) The genetics of division of labour in honey bee colonies. Advances in Insect Physiology 23: 117–169.

[17] Isidori AM, Buvat J, Corona G, Goldstein I, Jannini EA, et al (2014) A Critical Analysis of the Role of Testosterone in Erectile Function: From Pathophysiology to Treatment-A Systematic Review. European Urology 65: 99–112. doi: 10.1016/j.eururo.2013.08.048 24050791 10.1016/j.eururo.2013.08.048

[18] Tursi A, Papa A, Danese S (2015) Review article: the pathophysiology and medical management of diverticulosis and diverticular disease of the colon. Alimentary Pharmacology & Therapeutics 42: 664–684. 26202723 10.1111/apt.13322

[19] Martignoni ME (1964) Pathophysiology in the insect. Annual Review of Entomology 9: 179–204.

[20] vanEngelsdorp D, Evans JD, Saegerman C, Mullin C, Haubruge E, et al (2009) Colony Collapse Disorder: a descriptive study. PLoS ONE 4: e6481 doi: 10.1371/journal.pone.0006481 19649264 10.1371/journal.pone.0006481 PMC2715894

[21] Cornman RS, Tarpy DR, Chen Y, Jeffreys L, Lopez D, et al (2012) Pathogen Webs in Collapsing Honey Bee Colonies. PLoS One 7: e43562 doi: 10.1371/journal.pone.0043562 22927991 10.1371/journal.pone.0043562 PMC3424165

[22] Cox-Foster DL, Conlan S, Holmes EC, Palacios G, Evans JD, et al (2007) A metagenomic survey of microbes in honey bee colony collapse disorder. Science 318: 283–287. doi: 10.1126/science.1146498 17823314 10.1126/science.1146498

[23] vanEngelsdorp D, Cox-Foster D, Frazier M, Ostiguy N, Hayes J (2007) “Fall-Dwindle Disease”: Investigations into the causes of sudden and alarming colony losses experienced by beekeepers in the fall of 2006.

[24] vanEngelsdorp D, Evans JD, Donovall L, Mullin C, Frazier M, et al (2009) "Entombed Pollen": A new condition in honey bee colonies associated with increased risk of colony mortality. Journal of Invertebrate Pathology 101: 147–149. doi: 10.1016/j.jip.2009.03.008 19361513 10.1016/j.jip.2009.03.008

[25] Lacey LA, Brooks W (1997) Initial handling and diagnosis of diseased insects In: Lacey LA, editor. Manual of techniques in insect pathology. pp. 1–16.

[26] Pennsylvania State University College of Agricultural Sciences, Mid-Atlantic Apiculture Research and Extension Consortium, United_States_Department_of_Agriculture (2011) A field guide to honey bees and their maladies. Pennsylvania State University.

[27] Ritter W, Akratanakul P (2006) Honey bee diseases and pests: a practical guide. Food and Agriculture Organization of the United Nations (FAO).

[28] Shimanuki H, Knox DA (2000) Diagnosis of Honey Bee Diseases, Agriculture Handbook No. AH–690. U.S. Department of Agriculture pp. 61.

[29] Spivak M, Reuter GS (2016) Honey Bee Diseases and Pests, A Companion to Beekeeping in Northern Climates Department of Entomology and Minnesota Extension Service, University of Minnesota pp. 34.

[30] The National Bee Unit (2015) Common Pests, Diseases and Disorders of the Adult Honey Bee. United Kingdom: The Animal and Plant Health Agency, Department for Environment, Food & Rural Affairs pp. 20.

[31] Sammataro D, de Guzman L, George S, Ochoa R, Otis G (2015) Standard methods for tracheal mite research. Journal of Apicultural Research 52: 1–20.

[32] Sammataro D (2006) An easy dissection technique for finding the tracheal mite,Acarapis woodi(Rennie) (Acari: Tarsonemidae), in honey bees, with video link. International Journal of Acarology 32: 339–343.

[33] World Organization for Animal Health (OIE) (2016) Chapter 2.2.4. Nosemosis of honey bees Manual of Diagnostic Tests and Vaccines for Terrestrial Animals: World Organization for Animal Health (OIE)

[34] Dade HA (1994) Anatomy and Dissection of the Honeybee. Cardiff, UK: International Bee Research Association.

[35] Snodgrass RE (1956) Anatomy of the Honey Bee. Ithaca, NY: Comstock Pub. Associates 334 p.

[36] Carreck NL, Andree M, Brent CS, Cox-Foster D, Dade HA, et al (2015) Standard methods forApis melliferaanatomy and dissection. Journal of Apicultural Research 52: 1–40.

[37] Pettis JS, Delaplane KS (2010) Coordinated responses to honey bee decline in the USA. Apidologie 41: 256–263.

[38] González-Santoyo I, Córdoba-Aguilar A (2012) Phenoloxidase: a key component of the insect immune system. Entomologia Experimentalis et Applicata 142: 1–16.

[39] Erickson E, Cohen A, Brummett D, Lusby W, Cameron B (1997) Tyrosine nodules in the gasters of adult honeybees. Journal of Invertebrate Pathology 70: 27–32.

[40] Bedick JC, Tunaz H, Aliza ARN, Putnam SM, Ellis MD, et al (2001) Eicosanoids act in nodulation reactions to bacterial infections in newly emerged adult honey bees, Apis mellifera, but not in older foragers. Comparative Biochemistry and Physiology C-Toxicology & Pharmacology 130: 107–117. 10.1016/s1532-0456(01)00226-5 11544147

[41] Trappmann W (1923) Die Bildung der peritrophischen Membran bei Apis mellifica L. Arch für Bienenk 5: 190–212.

[42] Weil E (1935) Vergleichend-morphologische Untersuchungen am Darmkanal einiger Apiden und Vespiden. Zeitschr Wiss Biol Abt a Zeitschr Morph U Okol Tiere 30: 438–478.

[43] Hertig M (1923) The normal and pathological histology of the ventriculus of the honeybee, with special reference to infection with Nosema apls. Journal of Parasitology Urbana 9: 109–140.

[44] Maiolino P, Iafigliola L, Rinaldi L, De Leva G, Restucci B, et al (2014) Histopathological findings of the midgut in European honey bee (Apis Mellifera L.) naturally infected by Nosema spp. Veterinary Medicine and Animal Sciences 2: 4.

[45] Peng Y, Baer-Imhoof B, Millar AH, Baer B (2015) Consequences of Nosema apis infection for male honey bees and their fertility. Sci Rep 5: 10565 doi: 10.1038/srep10565 26123530 10.1038/srep10565 PMC4485221

[46] Snow JW (2016) A fluorescent method for visualization of Nosema infection in whole-mount honey bee tissues. J Invertebr Pathol 135: 10–14. doi: 10.1016/j.jip.2016.01.007 26802732 10.1016/j.jip.2016.01.007

[47] White GF (1919) Nosema-disease. Bulletin United States Department of Agriculture 780: (1–59).

[48] Dow JAT (1986) Insect midgut function. Advances in Insect Physiology 19: 187–328.

[49] Kummel G, and Zerbst-Boroffka I. (1974) Electron microscopic and physiological studies on the rectal pads in Apis mellifica. Cytobiology 9: 432–459.

[50] Lothmar R (1946) Über Flagellaten und Bakterien im Dünndarm der Honigbiene (*Apis mellifica*). Beihefte zur Schweizerischen Bienen-Zeitung: 49–76.

[51] Engel P, Bartlett KD, Moran NA (2015) The Bacterium Frischella perrara Causes Scab Formation in the Gut of its Honeybee Host. MBio 6. 10.1128/mBio.00193-15 PMC4442143 25991680

[52] Dow JA, Davies SA (2006) The Malpighian tubule: rapid insights from post-genomic biology. J Insect Physiol 52: 365–378. doi: 10.1016/j.jinsphys.2005.10.007 16310213 10.1016/j.jinsphys.2005.10.007

[53] Liu TP (1985) Scanning electron-microscope observations on the pathological changes of malpighian tubules in the worker honeybee, Apis mellifera, infected by maphiamoeba-mellificae. Journal of Invertebrate Pathology 46: 125–132.

[54] Sohal RS, Peters PD, Hall TA (1976) Fine structure and X-ray microanalysis of mineralized concretions in Malpighian tubules of the housefly, Musca domestica. Tissue & Cell 8: 447–458. 982422 10.1016/0040-8166(76)90005-7

[55] Beyenbach KW (2003) Transport mechanisms of diuresis in Malpighian tubules of insects. Journal of Experimental Biology 206: 3845–3856. 14506220 10.1242/jeb.00639

[56] Gramigni E, Calusi S, Chelazzi G, Del Greco F, Delfino G, et al (2011) Analysis of metal deposit distribution in ants (Crematogaster scutellaris) at the Florence external scanning microbeam. X-Ray Spectrometry 40: 186–190.

[57] Corby-Harris V, Maes P, Anderson KE (2014) The Bacterial Communities Associated with Honey Bee (*Apis mellifera*) Foragers. PLoS ONE 9: e95056 doi: 10.1371/journal.pone.0095056 24740297 10.1371/journal.pone.0095056 PMC3989306

[58] Kačániová M, Chlebo R, Kopernický M, Trakovická A (2004) Microflora of the honeybee gastrointestinal tract. Folia Microbiologica 49: 169–171. 15227790 10.1007/BF02931394

[59] Rada V, Machova M, Huk J, Marounek M, Duskova D (1997) Microflora in the honeybee digestive tract: counts, characteristics and sensitivity to veterinary drugs. Apidologie 28: 357–365.

[60] Forsgren E, Olofsson TC, Vasquez A, Fries I (2010) Novel lactic acid bacteria inhibiting Paenibacillus larvae in honey bee larvae. Apidologie 41: 99–108.

[61] Ahn J-H, Hong I-P, Bok J-I, Kim B-Y, Song J, et al (2012) Pyrosequencing analysis of the bacterial communities in the guts of honey bees Apis cerana and Apis mellifera in Korea. Journal of Microbiology 50: 735–745. 10.1007/s12275-012-2188-0 23124740

[62] Nation J (2005) Alimentary Canal and Digestion Encyclopedia of Entomology: Springer Netherlands pp. 65–71.

[63] Graham JM, Ambrose JT, Langstroth LL, Dadant & Sons (1992) The Hive and the Honey Bee; Graham JM, editor. Hamilton, Ill: Dadant xxv, 1324 p. p.

[64] Fyg W (1964) Anomalies and diseases of queen honey bee. Annual Review of Entomology 9: 207–224.

[65] Gurvits GE, Lan G (2014) Enterolithiasis. World J Gastroenterol 20: 17819–17829. 25548480 10.3748/wjg.v20.i47.17819 PMC4273132

[66] Calderone NW, Page RE Jr., (1988) Genotypic Variability in Age Polyethism and Task Specialization in the Honey Bee, Apis-Mellifera (Hymenoptera, Apidae). Behav Ecol Sociobiol 22: 17–25.

[67] Page RE Jr., Waddington KD, Hunt G, Fondrk MK(1995) Genetic determinants of honey bee foraging behaviour. Anim Behav 50: 1617–1625.

[68] Fyg W (1960) Über krankhafte Steinbildung im Rektum der Bienenkönigin. Zeitschrift für Bienenkunde 5: 93–100.

[69] Hillyer JF, Estévez-Lao TY (2010) Nitric oxide is an essential component of the hemocyte-mediated mosquito immune response against bacteria. Developmental & Comparative Immunology 34: 141–149. 19733588 10.1016/j.dci.2009.08.014

[70] Miller J, Chi T, Kapahi P, Kahn AJ, Kim MS, et al (2013) Drosophila melanogaster as an Emerging Translational Model of Human Nephrolithiasis. The Journal of Urology 190: 1648–1656. doi: 10.1016/j.juro.2013.03.010 23500641 10.1016/j.juro.2013.03.010 PMC3842186

[71] Evans JM, Allan AK, Davies SA, Dow JA (2005) Sulphonylurea sensitivity and enriched expression implicate inward rectifier K+ channels in Drosophila melanogaster renal function. J Exp Biol 208: 3771–3783. doi: 10.1242/jeb.01829 16169954 10.1242/jeb.01829

[72] McGettigan J, McLennan RK, Broderick KE, Kean L, Allan AK, et al (2005) Insect renal tubules constitute a cell-autonomous immune system that protects the organism against bacterial infection. Insect Biochem Mol Biol 35: 741–754. doi: 10.1016/j.ibmb.2005.02.017 15894191 10.1016/j.ibmb.2005.02.017

[73] Dow JA (2009) Insights into the Malpighian tubule from functional genomics. J Exp Biol 212: 435–445. doi: 10.1242/jeb.024224 19151219 10.1242/jeb.024224

[74] Satyavathi VV, Minz A, Nagaraju J (2014) Nodulation: An unexplored cellular defense mechanism in insects. Cellular Signalling 26: 1753–1763. doi: 10.1016/j.cellsig.2014.02.024 24704121 10.1016/j.cellsig.2014.02.024

[75] Aguila JR, Suszko J, Gibbs AG, Hoshizaki DK (2007) The role of larval fat cells in adult Drosophila melanogaster. Journal of Experimental Biology 210: 956–963. doi: 10.1242/jeb.001586 17337708 10.1242/jeb.001586

[76] Santos DE, Azevedo DO, Oliveira Campos LA, Zanuncio JC, Serrao JE (2015) Melipona quadrifasciata (Hymenoptera: Apidae) fat body persists through metamorphosis with a few apoptotic cells and an increased autophagy. Protoplasma 252: 619–627. doi: 10.1007/s00709-014-0707-z 25269629 10.1007/s00709-014-0707-z

[77] Nicolson SW (2009) Water homeostasis in bees, with the emphasis on sociality. J Exp Biol 212: 429–434. doi: 10.1242/jeb.022343 19151218 10.1242/jeb.022343

[78] Schmickl T, Crailsheim K (2004) Inner nest homeostasis in a changing environment with special emphasis on honey bee brood nursing and pollen supply. Apidologie 35: 249–263.

[79] R Core Team (2014) R: A language and environment for statistical computing 3.0.3 ed Vienna, Austria: R Foundation for Statistical Computing.

[80] Bates D, Maechler M, Bolker B, Walker S (2014) lme4: Linear mixed-effects models using Eigen and S4. 1.1–7 ed.

[81] Christensen RHB (2013) ordinal—Regression models for ordinal data. 2013.9–30 ed.

[82] Hothorn T, Bretz F, Westfall P, Heiberger RM, Schuetzenmeister A, et al. (2015) Simultaneous Inference in General Parametric Models. 1.4–0 ed. 10.1002/bimj.200810425 18481363

[83] Breiman L, Friedman J, Stone CJ, Olshen RA (1984) Classification and regression trees: CRC press.

[84] Saegerman C, Alba-Casals A, García-Bocanegra I, Dal Pozzo F, Galen G (2014) Clinical sentinel surveillance of equine West Nile fever, Spain. Transboundary and emerging diseases. 10.1111/tbed.12243 24899369

[85] Saegerman C, Porter SR, Humblet MF (2011) The use of modelling to evaluate and adapt strategies for animal disease control. Revue Scientifique Et Technique-Office International Des Epizooties 30: 555–569. 10.20506/rst.30.2.2048 21961226

[86] Saegerman C, Speybroeck N, Dal Pozzo F, Czaplicki G (2015) Clinical indicators of exposure to Coxiella burnetii in dairy herds. Transboundary and emerging diseases 62: 46–54. doi: 10.1111/tbed.12070 23480126 10.1111/tbed.12070

[87] Zanella G, Martinelle L, Guyot H, Mauroy A, De Clercq K, et al (2013) Clinical Pattern Characterization of Cattle Naturally Infected by BTV‐8. Transboundary and emerging diseases 60: 231–237. doi: 10.1111/j.1865-1682.2012.01334.x 22571462 10.1111/j.1865-1682.2012.01334.x

[88] Porter RS, Leblond A, Lecollinet S, Tritz P, Cantile C, et al (2011) Clinical Diagnosis of West Nile Fever in Equids by Classification and Regression Tree (CART) Analysis and Comparative Study of Clinical Appearance in Three European Countries. Transboundary and Emerging Diseases 58: 197–205. doi: 10.1111/j.1865-1682.2010.01196.x 21208395 10.1111/j.1865-1682.2010.01196.x

