# Supplementary material for: Colony Collapse Disorder (CCD) and bee age impact honey bee pathophysiology
Source: PLoS One. 2017 Jul 17;12(7):e0179535. doi: 10.1371/journal.pone.0179535 (PMC5513415; doi:10.1371/journal.pone.0179535)
Supplement: S1 Fig — (PDF) [file pone.0179535.s001.pdf]

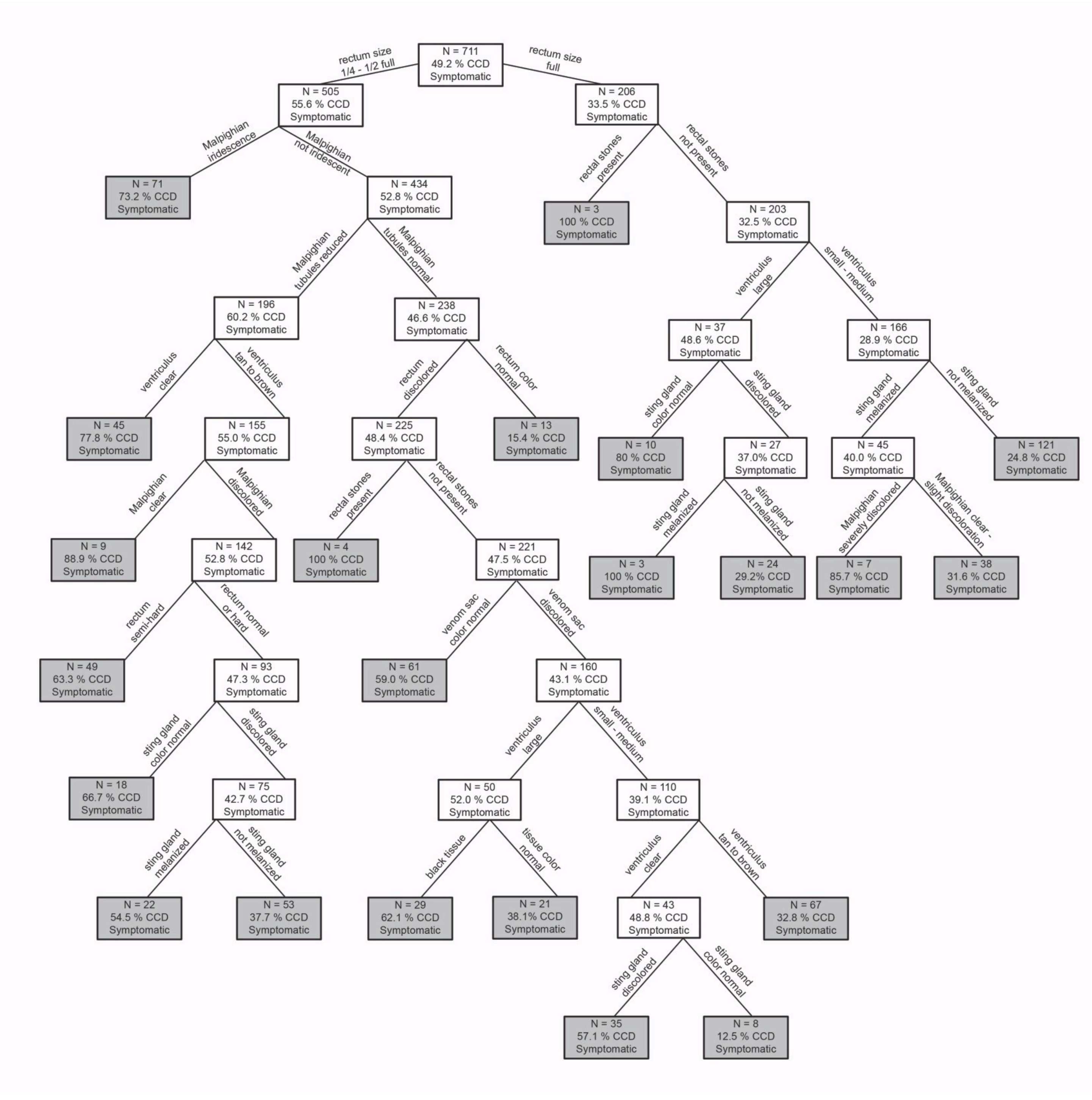

Figure S1, CART analysis of bees from CCD symptomatic (n = 350) and non-symptomatic colonies (n = 361) from all investigated apiaries. Boxes shaded in grey indicate a terminal node, where the decision tree ends. This classification tree had a specificity of 59.3% (95% CI: 54.0-64.4) and a sensitivity of 60.3% (95% CI: 54.9-65.4).
